# Perceptions, Reporting, and Responses to Depression Among Black Sub-Saharan African Immigrant Adults in the United States: A Scoping Review

**DOI:** 10.3390/nursrep16060196

**Published:** 2026-06-08

**Authors:** Kechi Iheduru-Anderson, Christiana O. Akanegbu, Chimezie J. Agomoh, Roop C. Jayaraman

**Affiliations:** 1 School of Rehabilitation and Medical Sciences, The Herbert H. and Grace A. Dow College of Health Professions, Central Michigan University, 1280 E Campus Drive, Mount Pleasant, MI 48859, USA; jayar1rc@cmich.edu; 2 Maureen Murphy Wilkens School of Nursing & Clinical Sciences, Emmanuel College, 400 The Fenway, Boston, MA 02115, USA; akanegbuc@emmanuel.edu; 3 School of Nursing and Health Sciences, Curry College, 1071 Blue Hill Ave, Milton, MA 02186, USA; cagomoh2009@curry.edu

**Keywords:** depression, Sub-Saharan African immigrants, Black immigrants, mental health perceptions, cultural conceptualization, somatization, scoping review

## Abstract

**Background:** Black Sub-Saharan African immigrants are among the fastest-growing immigrant populations in the United States, and their mental health needs, particularly with respect to depression, remain understudied. Cultural beliefs, linguistic frameworks, and coping practices in this population often diverge from Western psychiatric models, suggesting that conventional approaches may fail to capture how distress is experienced and expressed. **Objective:** This scoping review mapped literature on how Black Sub-Saharan African immigrant adults in the United States perceive, report, and respond to depression. **Methods:** Following PRISMA-ScR guidelines, six electronic databases were systematically searched for empirical studies published between 2000 and 2026. Two reviewers independently screened and extracted data using a standardized form. Data were analyzed using a narrative synthesis approach combining deductive thematic categorization across three predefined review domains with inductive identification of subthemes through iterative team discussion and consensus, with sociocultural, religious, linguistic, and structural factors examined as cross-cutting themes. Findings were synthesized narratively across three domains: perceptions of depression, reporting and communication, and responses to depression. **Results**: A total of 19 studies met the inclusion criteria (7 quantitative, 10 qualitative, 2 mixed methods; total N ≈ 1900), generating 24 themes. Perception themes highlighted cultural non-recognition of depression (12 of 19 studies), absence of equivalent terms in African languages (7 studies), spiritual explanatory models, and profound stigma. Reporting patterns showed predominant somatic symptom expression and very low disclosure to providers (2.6–4.2%), with depression prevalence ranging from 8.1% to 100% and no validated screening instrument identified for this population. Response themes emphasized religion and social support as primary coping strategies, with formal mental health utilization virtually absent due to structural, cultural, and intersectional barriers. **Conclusions:** Depression among Black Sub-Saharan African immigrants is widely experienced yet rendered invisible through interlocking cultural, linguistic, somatic, and institutional mechanisms, which this review terms an *architecture of invisibility*, leaving it largely unaddressed by formal mental health systems. The identification of only one intervention study underscores a substantial gap between documenting the burden of depression and advancing evidence-informed solutions. Culturally validated measures, faith-based intervention models, longitudinal designs, and attention to structural determinants are urgently needed.

## 1. Introduction

Depression is a leading cause of disability worldwide, affecting more than 280 million people globally [1,2]. The World Health Organization recognizes depression as a significant public health concern that transcends geographic, cultural, and socioeconomic boundaries [2]. The global burden of mental disorders, particularly depression and anxiety, continues to increase, with projections indicating that mental health disorders will become the largest contributor to global disease burden by 2030 [1,3]. Yet this burden is unevenly distributed: substantial disparities in recognition, diagnosis, treatment access, and outcomes persist among marginalized and immigrant communities, including Black Sub-Saharan African immigrants.

The Black Sub-Saharan African immigrant population in the United States has grown substantially over the past three decades, representing one of the fastest-growing immigrant groups in the country [4]. Between 2000 and 2019, this population more than doubled, with current estimates exceeding 2.1 million individuals, representing approximately 5% of the U.S. foreign-born population [5]. These immigrants originate from diverse nations, including Nigeria, Ethiopia, Ghana, Kenya, Somalia, Liberia, and others, bringing varied linguistic, cultural, religious, and socioeconomic backgrounds [4]. Despite this diversity, they often face shared experiences of migration-related stress, acculturation challenges, discrimination, and systemic barriers to healthcare access. Notably, even with higher educational attainment compared to other immigrant groups, many Black Sub-Saharan African immigrants experience economic marginalization and underemployment conditions that can heighten vulnerability to depression [6].

Black Sub-Saharan African immigrants encounter multiple, intersecting factors that influence their mental health and access to care. The migration experience itself can be traumatic, particularly for refugees and asylum seekers who may have experienced conflict, persecution, or displacement [7,8]. Post-migration stressors include acculturation difficulties, loss of social support networks, family separation, underemployment despite high educational attainment, experiences of racism and xenophobia, and navigating unfamiliar healthcare systems [9]. These cumulative stressors place immigrants at elevated risk for depression and other mental health conditions, with some studies reporting depression prevalence rates as high as 44% among first-generation African migrants [10].

Additionally, this population faces the compounding effects of being both Black and immigrant in the United States, experiencing what scholars describe as ‘double jeopardy’ or intersectional discrimination [11]. Research suggests that Black immigrants may face discrimination based on both race and immigrant status, which can exacerbate mental health challenges while simultaneously creating barriers to seeking and receiving appropriate care [12].

Cultural conceptualizations of mental illness vary significantly across Sub-Saharan African communities and often differ markedly from Western biomedical models of depression [13]. Cultural understandings of mental distress often diverge from Western biomedical models, emphasizing spiritual, relational, or somatic explanations rather than psychological constructs. [14]. Depression may be attributed to spiritual affliction, witchcraft, family curses, or imbalances in social harmony, leading individuals to seek help from traditional healers, religious leaders, or family elders rather than mental health professionals [15,16].

Stigma surrounding mental illness remains pervasive in many Sub-Saharan African communities, where depression may be viewed as a sign of personal weakness, spiritual failing, or family shame [17,18]. Such stigma contributes to under-reporting of symptoms, delayed help-seeking, and reluctance to engage with formal mental health services. Importantly, stigma manifests in gender-specific ways. Black immigrant women report elevated stigma concerns related to seeking mental health care and to being labeled as having a mental illness, which significantly affects their willingness to engage with formal services [17,19]. Black immigrant men, by contrast, face heightened stigma around the acknowledgment and expression of emotional vulnerability itself, with cultural masculinity norms discouraging the disclosure of psychological distress [17]. These two patterns reflect distinct dimensions of mental health stigma rather than conflicting findings: women’s stigma concerns are oriented toward help-seeking and social judgment, whereas men’s stigma concerns are oriented toward emotional expression and the cultural permissibility of vulnerability. Black immigrant women, in particular, report higher stigma concerns that significantly impact their willingness to seek mental health care [17,19]. Additionally, many Sub-Saharan African languages lack direct equivalents for the Western concept of “depression,” which can complicate symptom recognition and communication with healthcare providers [18].

Religious and spiritual beliefs play central roles in how many Black Sub-Saharan African immigrants understand and respond to emotional distress [14]. Faith-based coping strategies, including prayer, religious community support, and consultation with spiritual leaders, are often preferred first-line responses to psychological suffering [15]. While these strategies can provide meaningful support, they may also delay or substitute for evidence-based mental health treatment [16].

Cultural values emphasizing collectivism, family honor, and emotional restraint may further discourage open discussion of mental health struggles [13]. The expectation to maintain composure and avoid burdening others with personal problems can lead individuals to suffer silently rather than seeking help. Gender roles and expectations also shape how depression is perceived and expressed, with men potentially facing greater stigma around emotional vulnerability [17].

Beyond cultural factors, Black Sub-Saharan African immigrants face substantial structural barriers to mental health care. With the current U.S. healthcare systems, common barriers include lack of health insurance, financial constraints, language discordance, limited familiarity, scarcity of culturally competent providers, transportation difficulties, and competing demands such as employment and caregiving responsibilities [20,21,22]. Immigration status concerns, including fears about public charge rules or deportation, may further deter help-seeking even among those who recognize their need for care. Research demonstrates that African migrants consistently show low utilization rates of mental health services despite high rates of psychological distress [10,23].

When immigrants do access mental health services, they may encounter providers who lack cultural competence or awareness of migration-related stressors [20]. Misunderstandings between patients and providers regarding symptom presentation, treatment expectations, and the role of family and community in healing can compromise care quality and therapeutic relationships. The tendency toward somatization—expressing psychological distress through physical symptoms—is common among African immigrants and often leads to missed diagnoses or inappropriate treatment [24]. The scarcity of mental health professionals from Sub-Saharan African backgrounds or with specialized training in immigrant mental health further limits access to culturally concordant care [20].

While existing literature has examined depression among various immigrant groups and documented mental health disparities affecting Black Americans, Black Sub-Saharan African immigrants remain an understudied population in mental health research [25]. The heterogeneity of this population—encompassing diverse ethnic groups, languages, religions, immigration pathways, and pre-migration experiences—adds complexity to understanding their mental health needs [26]. Existing studies often aggregate all Black immigrants into a single analytic category, conflating distinct populations with different experiences, or focus narrowly on specific refugee groups, limiting generalizability.

Furthermore, research examining how Black Sub-Saharan African immigrants themselves perceive, experience, and respond to depression remains limited [25]. Understanding these perspectives is essential for developing culturally responsive interventions, improving clinical practice, training healthcare providers, and informing policy initiatives aimed at reducing mental health disparities. A comprehensive mapping of existing evidence regarding perceptions, reporting patterns, and help-seeking responses to depression in this population is needed to identify what is known, reveal critical gaps, and guide future research priorities.

### 1.1. Rationale for a Scoping Review

A scoping review methodology is particularly appropriate for examining depression among Black Sub-Saharan African immigrant adults in the United States for several reasons [27,28,29]. First, the scope of this topic, encompassing perceptions, reporting behaviors, and responses to depression, necessitates an exploratory approach that accommodates varied study designs, thereby capturing the breadth of current knowledge. Second, the potentially limited and scattered nature of existing research on this specific population necessitates a comprehensive search to determine the extent and nature of available evidence. Third, a scoping review can accommodate diverse study designs and methodologies, including qualitative studies exploring lived experiences, quantitative studies examining prevalence or patterns, and mixed-methods research, thereby capturing the full scope of available knowledge [29].

This scoping review will systematically map the existing literature on how Black Sub-Saharan African immigrant adults in the United States perceive, report, and respond to depression. By synthesizing current evidence, identifying knowledge gaps, and clarifying key concepts and frameworks in this area of research, this review will provide a foundation for developing targeted interventions, guiding clinical practice, and establishing research priorities to address mental health disparities in this growing, underserved population. The review will follow the PRISMA Extension for Scoping Reviews (PRISMA-ScR) guidelines to ensure transparent and comprehensive reporting [30].

### 1.2. Review Objectives

The objectives of this scoping review are to:

1. Identify and map the existing literature on perceptions of depression among Black Sub-Saharan African immigrant adults in the United States.
2. Examine how depression is reported or communicated by this population in research and clinical contexts.
3. Characterize the range of responses to depression, including help-seeking behaviors, coping strategies, and patterns of treatment engagement.
4. Identify sociocultural, linguistic, religious, and structural factors that shape depression recognition and response in this population.
5. Determine gaps in the current evidence base and propose priorities for future research.

### 1.3. Research Question

How do Black Sub-Saharan African immigrant adults in the United States perceive, report and respond to depression?

## 2. Methods

### 2.1. Study Design

This scoping review was conducted following the methodological framework proposed by Arksey and O’Malley [27], as subsequently refined by Levac et al. [31] and the Joanna Briggs Institute guidance [29]. The review adhered to the Preferred Reporting Items for Systematic Reviews and Meta-Analyses Extension for Scoping Reviews (PRISMA-ScR) reporting guidelines [30]. The protocol was registered with the Open Science Framework upon completion of the review and prior to journal submission (registration DOI: https://osf.io/g2aun; registered 9 April 2026). As this registration was completed retrospectively, it serves as a transparent record of the methods employed rather than a prospective preregistration.

### 2.2. Search Strategy

#### 2.2.1. Information Sources

A comprehensive literature search was conducted across six electronic databases from January 2000 to January 2026: PubMed, PsycINFO, CINAHL, Web of Science, Scopus, and Sociological Abstracts. Supplementary searches of ProQuest Dissertations and Theses Global and Google Scholar were conducted to identify grey literature. Reference lists of included studies and relevant reviews were hand-searched, and forward and backward citation tracking was performed. These supplementary searches yielded no additional studies beyond those identified through database searching.

#### 2.2.2. Search Terms

The search strategy combined three concept blocks using Boolean operators: (1) Sub-Saharan African immigrants (including specific country names and terms such as “African immigrant,” “African refugee,” and “Black African”); (2) depression and mental health; and (3) perceptions, help-seeking, barriers, reporting, and responses. All terms were further combined with “United States” to specify geographic context. Medical Subject Headings (MeSH) and database-specific controlled vocabulary were utilized where applicable. The search strategy was developed in consultation with a health sciences librarian and pilot-tested before full implementation. The complete search strategy for all databases is provided in Supplementary File S1.

#### 2.2.3. Eligibility Criteria

Studies were included if they met all of the following criteria: (1) focused on Black Sub-Saharan African immigrant or refugee adults (≥18 years) residing in the United States; (2) addressed depression, major depressive disorder, or depressive symptoms; (3) examined at least one of the following domains: perceptions or beliefs about depression, reporting or communication of depressive symptoms, or responses to depression, including help-seeking behaviors, coping strategies, treatment engagement, and barriers or facilitators to care; (4) primary empirical research using qualitative, quantitative, or mixed-methods designs; (5) were published in English; and (6) were published between January 2000 and the final search date.

Studies were excluded if they: (1) focused exclusively on children or adolescents (<18 years); (2) focused on African Americans (U.S.-born individuals of African descent) without specific examination of immigrant experiences; (3) focused on North African (e.g., Egypt, Morocco) or Caribbean (e.g., Jamaica, Haiti) populations; (4) included Sub-Saharan African immigrants as part of a mixed sample without providing disaggregated data; (5) were conducted outside the United States; (6) examined only depression prevalence without addressing perceptions, reporting, or responses; (7) were review articles, editorials, commentaries, opinion pieces, or conference abstracts that lacked sufficient methodological detail; or (8) were published in languages other than English.

The January 2000 cutoff was selected because the contemporary growth of the Black Sub-Saharan African immigrant population in the United States is largely a post-2000 phenomenon. According to U.S. Census and Pew Research Center data, this population more than doubled between 2000 and 2019 [5], making the post-2000 era the period most relevant to characterizing current immigrant experiences, healthcare system interactions, and policy contexts. The Diversity Visa Lottery and refugee resettlement patterns that shape much of the current Sub-Saharan African immigrant community in the United States are also concentrated in this period. Studies published prior to 2000 would predominantly describe immigrant cohorts that differ substantially in size, composition, and contextual circumstances from the contemporary population of interest.

North African (e.g., Egyptian, Moroccan, Tunisian, Libyan, Algerian) and Caribbean (e.g., Jamaican, Haitian, Trinidadian) populations were excluded because they have distinct cultural, linguistic, religious, and migration contexts that differ substantially from those of Sub-Saharan African immigrants. North African populations are most often classified within the Middle East and North Africa (MENA) region and are characterized by predominantly Arabic linguistic traditions and Islamic religious heritage, with distinct cultural frameworks for understanding mental illness that are addressed in a separate body of literature. Caribbean populations include Afro-Caribbean immigrants whose cultural and linguistic heritage reflects post-colonial Caribbean creole traditions, distinct migration histories, and different patterns of integration into U.S. Black communities, also addressed in a separate body of literature. Both populations have been the subject of dedicated mental health reviews and aggregating them with Sub-Saharan African immigrants would dilute the cultural specificity central to this review’s objectives.

#### 2.2.4. Study Selection

All retrieved records were imported into Covidence systematic review software (Veritas Health Innovation, Melbourne, Australia; web-based platform with no version number). The database search identified 1182 records (CINAHL, n = 752; Web of Science, n = 220; PubMed, n = 104; Scopus, n = 41; PsycINFO, n = 35; Sociological Abstracts, n = 30). Supplementary citation searching and grey literature searches yielded no additional records. Following deduplication (417 duplicates identified by Covidence and 7 identified manually), 424 records were removed, leaving 758 records for title and abstract screening. Prior to screening, the review team met to review the study objectives and the inclusion and exclusion criteria to ensure shared understanding and consistency. A pilot screening of 50 records was then conducted independently by two reviewers to calibrate the criteria application, yielding an inter-rater agreement of 87%. Remaining discrepancies were discussed and resolved by consensus before full screening commenced.

**Figure 1:**
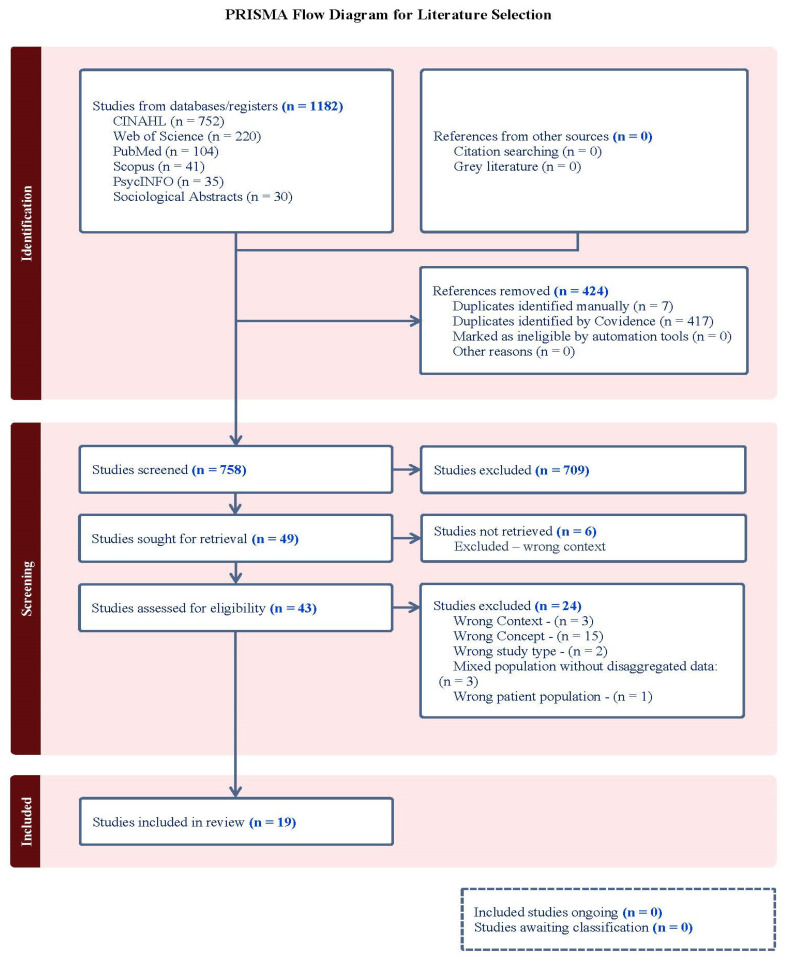
PRISMA Flow Diagram for Literature Selection.

After title and abstract screening, 709 records were excluded, and 49 studies were sought for full-text retrieval. Six records could not be retrieved and were excluded due to the wrong context, leaving 43 studies assessed for full-text eligibility. Two independent reviewers assessed all full texts, with conflicts resolved by a third reviewer. A total of 24 studies were excluded at this stage because of the wrong concept (n = 15), a mixed population without disaggregated data (n = 3), the wrong context (n = 3), the wrong study type (n = 2), or the wrong patient population (n = 1). Nineteen studies met all inclusion criteria and are included in this review. No ongoing studies or studies awaiting classification were identified. Reasons for exclusion at the full-text stage were documented, and the complete study selection process is depicted in a PRISMA-ScR flow diagram (Figure 1).

### 2.3. Data Extraction

A standardized data extraction form was developed in Microsoft Excel and organized across key domains aligned with the review objectives: study characteristics (author, year, location, setting, design); participant characteristics (sample size, age, gender, countries of origin, immigration status, time in U.S.); depression assessment methods; and key findings related to perceptions of depression (cultural conceptualizations, explanatory models, stigma), reporting patterns (symptom expression, communication, somatization), and responses to depression (help-seeking behaviors, coping strategies, treatment utilization, barriers and facilitators). Relevance ratings for included studies were determined through team review and consensus, with all reviewers contributing to final designations. The primary reviewer extracted data from all 19 included studies using the standardized extraction form. An artificial intelligence tool (Claude, Anthropic) was used exclusively for post-extraction organizational tasks, including formatting and structuring the already-extracted data into tabular displays, and played no role in data extraction, interpretation, or analytical judgment. A second and third reviewer independently verified all extracted data against the original source articles, and discrepancies were resolved through discussion by the team. The extraction form was iteratively refined during the early stages of data extraction to ensure that all relevant data fields were captured.

#### Relevance Rating Criteria

Each included study was assigned a relevance rating reflecting the degree to which it directly addressed the review’s three core domains (perceptions, reporting, and responses to depression) and provided substantive data relevant to the research question. Ratings were not based on a preset numerical count of themes but on a qualitative assessment of three criteria: (a) the centrality of Black Sub-Saharan African immigrants as the primary study population, (b) the breadth of domain coverage, that is, whether the study addressed one, two, or all three review domains substantively, and (c) the depth and richness of findings relevant to the research question. The rating categories and the criteria for distinguishing them are summarized in Table 1.

Ratings were determined through a full-team review and consensus, with each reviewer contributing an independent assessment before discussion. Where ratings diverged, the team discussed the study together and reached a consensus rating. This interpretive approach is consistent with scoping review methodology, in which the goal of relevance assessment is to characterize, rather than to numerically score, the contribution of each study to the evidence map.

### 2.4. Data Synthesis

A narrative synthesis approach was employed due to anticipated heterogeneity in study designs, populations, and outcomes [32]. Study characteristics, publication trends, and participant demographics were summarized using frequency counts, ranges, and tabular displays. Findings were organized through thematic categorization across three domains derived deductively from the research question: (1) perceptions and cultural conceptualizations of depression, (2) reporting and communication of depressive symptoms, and (3) responses to depression, including help-seeking behaviors and coping strategies. Within these domains, subthemes were identified inductively from the extracted data through iterative team discussion and consensus. Sociocultural, religious, linguistic, and structural factors influencing these domains were analyzed as cross-cutting themes.

Where findings were ambiguous or could plausibly belong to multiple thematic categories, the full review team met to discuss their placement, with final assignment determined by consensus. Findings that contradicted or complicated dominant patterns were deliberately retained and foregrounded in the synthesis rather than minimized, because divergent evidence was considered equally informative for mapping the breadth of the literature. A thematic mapping matrix was developed to visually display the distribution of themes across studies and identify patterns of convergence and divergence. Consistent with scoping review methodology [27,29], a formal quality assessment of individual studies was not conducted; however, methodological characteristics were noted and considered when interpreting the findings.

## 3. Results

### 3.1. Overview of Included Studies

The search strategy identified 1182 records across six databases. Following deduplication (n = 424) and title/abstract screening (n = 758 screened; n = 709 excluded), 49 studies were sought for full-text retrieval. Six records could not be retrieved. Of the 43 studies assessed, 24 were excluded, yielding 19 studies that met the inclusion criteria (See Table 2), published between 2010 and 2025. The included studies comprised 7 quantitative studies (S001, S002, S004, S011, S012, S018, S019), 10 qualitative studies (S003, S005, S006, S007, S008, S009, S010, S015, S016, S017), and 2 mixed-methods studies (S013, S014). The aggregate sample sizes totaled approximately 1900 participants, after accounting for overlapping samples between S001/S002 and S014/S017.

Geographically, studies were concentrated in the Pacific Northwest (6 studies from Seattle and Portland), followed by the Midwest (4 studies), Mid-Atlantic/Southeast (3 studies), South (2 studies from Houston), and Northeast (2 studies). Only one study (S019) employed a nationwide sample. Study populations most frequently included Somali refugees or immigrants (7 of 19 studies), followed by Nigerian immigrants (2 studies), Ethiopian immigrants (1 dedicated study plus representation in multi-country samples), and Congolese refugees (1 study). Five studies were sampled from multiple Sub-Saharan African countries. Central and Southern African populations were minimally represented.

Among the 9 studies employing standardized depression measures, instruments included the Hopkins Symptom Checklist-25 (HSCL-25; 4 studies), Patient Health Questionnaire-8 (PHQ-8; 3 studies), Patient Health Questionnaire-4 (PHQ-4; 1 study), and clinical DSM-IV diagnosis (1 study). Notably, no study used a depression measure validated specifically for Black African immigrant populations, a finding that has important implications for prevalence estimates across the literature.

The narrative synthesis that follows is organized around three primary domains aligned with the review question: (1) perceptions of depression, (2) reporting and communication of depression, and (3) responses to depression. Cross-cutting factors including gender, age, immigration status, discrimination, and acculturation are integrated throughout. Across all three domains, findings converge on a recurring pattern in which depression is rendered invisible through interlocking cultural, linguistic, somatic, and institutional mechanisms, a framework developed more fully in the Discussion. An evidence gap analysis concludes the synthesis. Table 3 displays the distribution of all 24 themes across included studies, indicating primary and secondary findings for each.

**Key observations:** Cultural barriers (C7) and stigma (P6) were the most widely documented themes, each appearing in 14–15 studies. The least documented themes were sociopolitical temporality (R5; 2 studies) and substance use as coping (C5; 2 studies). The three most thematically dense studies were S009 [41]; undocumented women), S008 [40]; Nigerian women), and S003 [35]; Somali case study).

### 3.2. Domain 1: Perceptions of Depression

Eight thematic areas emerged across the 19 studies regarding how Black Sub-Saharan African immigrants perceive depression. These themes were not mutually exclusive; rather, they formed an interconnected web of cultural, linguistic, spiritual, and social frameworks that collectively shaped whether and how depression was recognized as a distinct condition.

#### 3.2.1. Cultural Non-Recognition of Depression

One of the most pervasive findings across the included studies was that depression is widely unrecognized as a discrete health condition within many Sub-Saharan African immigrant communities. This theme emerged in 12 of 19 studies (S003, S004, S005, S006, S007, S008, S009, S010, S011, S014, S015, S016), spanning Somali, Nigerian, Congolese, Ethiopian, Liberian, and multi-country samples. The evidence suggests that cultural non-recognition operates through multiple, reinforcing mechanisms.

Among Nigerian immigrants, Ezeobele and colleagues conducted companion studies examining perceptions of depression among men (S007) and women (S008) in Houston, Texas. The overarching finding for men was captured in the theme “Depression does not exist in the Nigerian culture” (S007), whereas for women, the overarching theme was “Depression is Not Acceptable” (S008). Despite Nigeria being a male-dominant culture, the researchers found no meaningful gender differences in this fundamental stance of non-recognition, suggesting that cultural non-recognition of depression transcends gender roles. Both studies documented an endurance norm in which individuals were “trained to endure” rather than acknowledge emotional distress (S008).

In Somali communities, non-recognition was structured by what Boynton and colleagues (S003) described as a sanity-insanity binary (waalli), in which individuals were classified as either sane or insane with no intermediate category. Nakajima and colleagues (S016) confirmed this binary framework persisted among Somali elders in Minnesota, noting that depression falls into “an invisible middle that the framework cannot accommodate.” However, S016 also identified an important generational shift: while elders maintained the binary framework, younger Somali immigrants were beginning to recognize depression as a distinct condition, suggesting evolving perceptions within these communities.

Among Liberian immigrants, Ludwig and Reed (S015) [47] documented through ethnographic observation that community members “did not identify [their distress] as mental health problems” despite experiencing symptoms consistent with depression. Similarly, among East African immigrants with HIV, Lipira and colleagues (S014) [46] found that participants needed help “giving a name to what people are feeling,” indicating that the absence of a conceptual framework for depression was not limited to any single cultural group but appeared to be a cross-cutting phenomenon across diverse Sub-Saharan African communities.

#### 3.2.2. The “Naming Gap”: Linguistic and Conceptual Absence

Closely related to cultural non-recognition was the finding that multiple African languages lack direct equivalents for Western psychiatric concepts of depression, anxiety, or mental health. This “naming gap” was documented in 7 studies (S004, S005, S007, S008, S014, S015, S016) and emerged as a prominent cross-cutting theme in the review.

The most direct evidence came from Chirimwami and colleagues’ (S004) [36] community-based participatory research with diverse Sub-Saharan African immigrants in Portland, Oregon. During post-survey community consultations, Advisory Board members noted that “there are no words for mental health or anxiety in Swahili or Somali,” highlighting the linguistic absence of mental health concepts as a barrier to both measurement and recognition [36]. This linguistic absence was echoed by Ezeobele and colleagues (S008), who reported that Igbo-speaking Nigerian women noted they had “no word for it” when attempting to describe depression. Among Somali immigrants, Nakajima and colleagues (S016) found that “the term stress is new” as a concept, indicating that even broader distress terminology was linguistically unfamiliar.

The practical implications of this naming gap were captured by Corley and Sabri (S005) [37], whose participants with intimate partner violence experience explicitly recommended to “not say mental health” and instead use symptom-based language when communicating with African immigrant women about emotional distress. This finding suggests that the naming gap is not merely linguistic but conceptual, necessitating fundamentally different approaches to screening and clinical communication.

#### 3.2.3. Spiritual and Supernatural Explanatory Model

Five studies (S003, S007, S008, S010, S016) documented spiritual or supernatural frameworks for understanding symptoms that Western psychiatry would classify as depression. These explanatory models were culturally specific and varied by ethnic group.

In Somali communities, Boynton and colleagues (S003) [35] described the concept of *jinn* (spirit possession) as the primary explanatory model for the composite case patient’s distress, leading to Qu’ran reading at the mosque as the first-line treatment before any psychiatric referral. Nakajima and colleagues (S016) [48] extended this finding, documenting that among Somali elders, depression could be attributed to weak faith or spirit possession, revealing a double-edged relationship with religion where faith served as both a source of resilience and a mechanism of blame.

Among Nigerian immigrants, Ezeobele and colleagues (S008) [39,40] documented the *ogbanje* concept specific to Igbo cosmology, a water spirit or dual personality explanation for what Western psychiatry would classify as depression. Half of the Nigerian women interviewed endorsed traditional or fetish treatments including consultations with juju priests and the use of ritualistic objects such as ground animal parts and pendants. Among Ethiopian immigrants, Kassa and colleagues (S010) [42] similarly found that mental illness was commonly viewed through a supernatural or spiritual lens, with participants referencing a general lack of health literacy about psychiatric conditions.

#### 3.2.4. Stigma, Shame, and Social Consequences

Stigma associated with depression or mental illness was documented in 14 of 19 studies, making it the most widely reported perception-related finding. However, the nature and mechanisms of stigma varied across cultural groups and contexts.

The most detailed account of stigma was documented by Ezeobele and colleagues (S008), who described the Nigerian marriage investigation system in which families conduct background checks spanning multiple generations to identify any history of mental illness before approving marriages. One participant provided a concrete example traced back to 1947, illustrating how stigma operates intergenerationally through formal social mechanisms. This finding suggests that the consequences of depression disclosure extend far beyond the individual to affect the social and economic prospects of entire family networks.

In Somali communities, Boynton and colleagues (S003) [35] documented the stigmatizing label of *“the sick ones,”* which led families to avoid association with individuals perceived as mentally ill. Corley and Sabri (S005) captured a broader sentiment among multi-country African immigrant women: “If you went to see a therapist, there was something wrong with you.” Among Congolese refugees, DiClemente-Bosco and colleagues (S006) [38] found that community gossip and fear of being labeled *“crazy”* served as powerful deterrents to disclosing emotional distress. In East African HIV-positive communities, Lipira (S014) [46] and Nevin (S017) [49] documented a compounded dual stigma where mental health stigma was layered on top of HIV-related stigma, creating an intensified barrier to recognition and disclosure.

#### 3.2.5. Depression as Situational and Normalized

A subset of studies (S005, S007, S008, S009, S015, S016, S017) documented a pattern in which depression was understood not as a medical condition but as a rational, expected response to difficult circumstances. This framing effectively normalized depressive symptoms as an inherent feature of the immigrant experience rather than a treatable health condition.

Among undocumented African immigrant women, Olukotun and colleagues (S009) [41] found that women “coped with their sadness and viewed it as a normal part of their experiences,” attributing their distress entirely to structural causes including undocumented status, economic exploitation, and a hostile sociopolitical environment. Among Liberian immigrants, Ludwig and Reed (S015) [47] documented a geographic attribution in which participants linked their distress to “being in America,” with one participant’s ailments reportedly disappearing when visiting Liberia. Nigerian immigrant men (S007) framed depression as incompatible with cultural expectations, stating “We were not raised to be depressed,” effectively recasting a health condition as a personal or cultural failing.

This normalization pattern carries profound implications for screening and intervention. If depression is perceived not as an illness but as an expected consequence of circumstance, affected individuals may see no reason to seek professional help, instead viewing their distress as something to be endured. Seven of the ten qualitative studies documented this pattern, suggesting it is widespread across diverse Sub-Saharan African immigrant communities.

### 3.3. Domain 2: Reporting and Communication of Depression

The second domain examined how depression symptoms are expressed, communicated, and reported by Black Sub-Saharan African immigrants. Six themes emerged across the included studies, revealing systematic patterns that have important implications for clinical detection and research measurement.

#### 3.3.1. Somatic Symptom Emphasis

The most robust reporting-related finding, documented in nine studies across both qualitative and quantitative designs, was the predominance of somatic over psychological symptom expression. This triangulation across methods strengthens the evidence that somatization represents a genuine cultural pattern of depression expression in this population rather than a measurement artifact.

The strongest quantitative evidence came from Nkimbeng and colleagues (S018) [50], who found that among older African immigrants, somatic symptoms on the PHQ-8 were endorsed at approximately twice the rate of psychological symptoms. Item-level endorsement rates are presented in Table 4. Sleep disturbance and fatigue were endorsed at roughly twice the rate of depressed mood, with psychomotor changes endorsed least frequently. These findings provide direct, item-level evidence that depression in this population is expressed predominantly through physical complaints.

Qualitative studies provided rich contextual data that corroborated this quantitative pattern. Ezeobele and colleagues documented that both Nigerian men (S007) and women (S008) described depression in terms of headaches, body pain, fatigue, and sleeplessness rather than emotional states. Ludwig and Reed (S015) [47] observed an embodiment of distress in which Liberian immigrants experienced depression as a chronic disease, captured in the participant statement, “When you are here, you have high blood pressure.” Bentley et al. (2011) (S001) [33] provided mechanistic evidence through mediation analysis, demonstrating that somatization significantly mediated the relationship between trauma and depression in Somali refugees, explaining 9% of the variance in HSCL-25 depression scores. Lever and colleagues (S013) [45] found that headaches were the single most frequently endorsed symptom on the HSCL-25 among West African FGM/C survivors.

#### 3.3.2. Non-Disclosure and Systematic Underreporting

A striking pattern of non-disclosure and help-seeking avoidance emerged across the qualitative literature, with quantitative studies providing corroborating evidence of likely systematic underreporting. Two studies documented extraordinarily low rates of professional contact despite high symptom prevalence: among undocumented African immigrant women, only 1 of 24 (4.2%) had discussed mental health symptoms with any healthcare provider (S009), and among African immigrant women experiencing intimate partner violence, only 1 of 39 (2.6%) had done so (S005). In the clinical context, Kroll and colleagues (S011) found that 25% of Somali refugee patients denied experiencing depression despite having received a clinical diagnosis, compared to 0% of non-Somali comparison patients, providing the most direct quantitative evidence of active denial in the reviewed literature.

Non-disclosure was driven by multiple, intersecting factors. Olukotun and colleagues (S009) [41] found that undocumented women’s secrecy about their immigration status extended to emotional distress, as any institutional encounter—including healthcare visits—carried the risk of detection and deportation. Women reported being strategic about “what type of spaces they accessed and when,” a behavioral pattern that effectively precluded help-seeking. Among East African HIV-positive immigrants, Lipira and colleagues (S014) [46] found that confidentiality was so critical that participants would accept peer support facilitators “as long as you’re not from my country,” revealing the fine-grained calibration of disclosure decisions even within immigrant communities. This was further illuminated by Nevin and colleagues (S017) [49], who reported that “depression is not very talked about in the African community,” framing silence around depression as a community-level norm rather than an individual choice.

These qualitative findings suggest that quantitative prevalence estimates derived from self-report instruments may systematically undercount depression in this population. Multiple authors explicitly raised this concern (S001, S004, S009, S011, S018, S019), citing cultural stigma, social desirability bias, the absence of validated instruments for African immigrant populations, and the healthy immigrant effect as sources of potential underreporting.

#### 3.3.3. Sociopolitical Temporality of Depressive Symptoms

A novel finding, documented primarily by Olukotun and colleagues (S009) and supported by Corley and Sabri (S005) [37], was that the severity of depressive and anxiety symptoms among undocumented African immigrant women was not static but fluctuated with the sociopolitical climate. Women with longer U.S. stays reported varying levels of distress across different political administrations, with one participant stating, “When Obama was the president, I felt this sense of normalcy. The fear I feel now was not as pronounced” (S009).

Several specific features of the Obama-era policy environment (2009–2017) likely contributed to this experience. The Deferred Action for Childhood Arrivals (DACA) program, established in 2012, provided temporary deportation protection and work authorization for eligible undocumented young adults brought to the United States as children, indirectly signaling a more inclusive enforcement posture toward non-criminal undocumented residents. The administration also issued prosecutorial discretion guidance prioritizing enforcement against individuals with serious criminal histories rather than indiscriminate enforcement against undocumented residents, and announced (though ultimately enjoined) the Deferred Action for Parents of Americans (DAPA) program. The 2014 enforcement priority memoranda redirected resources away from long-term residents and toward recent border crossers and those with felony convictions. Public rhetoric during this period generally characterized the undocumented immigrant population in less stigmatizing terms, and major immigration raids in residential communities were less frequent.

In contrast, the subsequent administration (2017–2021) implemented expanded interior enforcement, public charge rule revisions that linked use of public benefits including Medicaid to immigration consequences, family separation at the border, the rescission attempt against DACA, and heightened anti-immigrant public rhetoric. Independent research has documented increases in psychological distress, healthcare avoidance, and chilling effects on benefit utilization among immigrant communities during this period. The participant’s account therefore reflects an empirically grounded difference in policy environment rather than a purely subjective impression.

The participant’s account therefore reflects an empirically grounded difference in policy environment rather than a purely subjective impression. This finding suggests that depression among undocumented African immigrant women is partly a product of the policy environment, challenging purely individual or cultural explanations and pointing to structural determinants of mental health that operate through fear of deportation, family separation risk, reduced access to safety-net services, and the cumulative chronic stress of an unpredictable enforcement climate.

#### 3.3.4. Prevalence Estimates Across Studies

Among the nine studies employing standardized depression measures, prevalence estimates ranged dramatically from 8.1% to 100%, reflecting heterogeneity in populations, measures, cutoffs, and sampling strategies. These data are summarized in Table 5.

The wide prevalence range reflects genuine population heterogeneity, with the lowest estimate among community-dwelling older adults and the highest among FGM/C survivors seeking asylum—a population with extreme trauma exposure. Intermediate estimates varied with population vulnerability and study setting. Notably, Chirimwami and colleagues (S004) reported that respondents endorsed mild depressive symptoms at significantly higher rates than both the general U.S. population and U.S. Non-Hispanic Black adults on 2022 national norms, with elevation concentrated in the mild rather than the moderate or severe category. The authors attributed this pattern in part to cultural differences in interpreting symptom questions and possible underreporting of moderate or severe symptoms, providing direct empirical support for the systematic underreporting concerns documented in the qualitative literature. The exceptionally high 94.7% prevalence reported by Leaman and Gee (S012) [44] warrants cautious interpretation, as this rate may reflect measurement characteristics or sample selection. Critically, the potential for systematic underreporting documented in the preceding section suggests that even these varied estimates may represent lower bounds of true depression prevalence.

### 3.4. Domain 3: Responses to Depression

The third domain examined how Black Sub-Saharan African immigrants responded to depression, encompassing coping strategies, help-seeking behaviors, treatment engagement, and the barriers and facilitators shaping these responses. Ten thematic areas emerged, organized below into coping and help-seeking patterns, barriers to formal care, and facilitators.

#### 3.4.1. Religious and Spiritual Coping

Religious or spiritual coping was the most frequently documented response to depression, identified in nine studies (S003, S005, S006, S007, S008, S009, S010, S012, S016). Across Somali, Nigerian, Ethiopian, Congolese, Liberian, and multi-country samples—and across both Christian and Muslim communities—religion served as a primary framework for managing emotional distress. However, the evidence revealed a critically important dual role.

As a source of resilience, religion provided hope, meaning, a sense of belonging, and a sense of purpose. Among undocumented women (S009), church volunteering gave participants “the purpose I needed. Just something to remind me that I am important,” directly countering the existential despair associated with their circumstances. Women framed their hardship as a transitional phase that God would resolve, providing a cognitive framework for endurance. Among Somali refugees (S003), Qur’an reading and mosque attendance served as culturally sanctioned first-line treatments. Among Nigerian immigrants, both men (S007) and women (S008) described prayer and church communities as central to coping.

However, Leaman and Gee (S012) [44] provided quantitative evidence that religious coping was significantly correlated with higher depression scores, suggesting it may also function as a mechanism of avoidance that delays professional help-seeking. Nakajima and colleagues (S016) documented this duality explicitly among Somali immigrants: while faith provided resilience, depression could simultaneously be attributed to weak faith or spirit possession, effectively blaming individuals for their illness and reinforcing non-disclosure. This dual role, simultaneously protective and potentially harmful, has significant implications for faith-based intervention design and must be carefully navigated.

#### 3.4.2. Social Support Networks

Social support was documented as a critical coping resource in eight studies (S003, S005, S006, S009, S010, S014, S015, S017), although the evidence also revealed that access to supportive networks was constrained by trust, immigration status, and intersectional marginalization.

Among undocumented women, Olukotun and colleagues (S009) [41] provided the most detailed account of how social networks were carefully constructed and calibrated around shared vulnerability. Women disclosed their status and distress only to “other Black people who are also undocumented,” describing a snowball-like process of network building through trusted referrals. This selective disclosure pattern was driven by the fear that “you’re struggling all by yourself. Nobody cares, it’s every man for himself.” Transnational support also played a role, with women calling family in their home countries for emotional support and receiving instrumental support, including food items and medications.

Quantitative evidence reinforced the protective role of social connectedness. Saasa and Miller (S019) [51] found that loneliness was the second strongest predictor of depression/anxiety (β = 0.37, *p* < 0.001), and more importantly, that loneliness significantly moderated the relationship between discrimination and mental health outcomes. When loneliness was high, discrimination’s negative impact was amplified; when social connectedness was present, it buffered the impact of discrimination. This moderation finding provides a mechanistic link between the isolation documented qualitatively and the depression outcomes measured quantitatively.

### 3.5. Formal Mental Health Service Utilization

Across the reviewed literature, formal mental health service utilization was remarkably low. The two studies documenting specific disclosure rates found that 4.2% of undocumented women (1 of 24; S009) and 2.6% of women experiencing IPV (1 of 39; S005) had discussed mental health symptoms with a healthcare professional. In the clinical setting, Kroll and colleagues (S011) found that even when Somali refugees were referred to psychiatric care, 25% denied experiencing depression. Only one study (S014) tested a formal mental health intervention in an African immigrant population, and only one participant in the entire qualitative literature (S009) had been considered for psychopharmacological treatment (Prozac). This pattern of near-absent formal help-seeking appears to be shaped by a multilayered system of barriers that can be categorized as structural, cultural, and intersectional.

#### 3.5.1. Structural Barriers

Structural barriers were documented in 11 studies and included lack of health insurance, inability to afford care, language barriers, transportation challenges, absence of culturally competent services, and the exclusion of undocumented immigrants from social safety net programs. These barriers were not merely inconveniences; they represented systemic exclusion from the healthcare system. Olukotun and colleagues (S009) [41] documented how undocumented women feared that any healthcare encounter could lead to detection and deportation, effectively rendering the healthcare system inaccessible regardless of their theoretical eligibility for safety-net services.

#### 3.5.2. Cultural Barriers

Cultural barriers were the most widely documented, appearing in 15 of 19 studies. These included the cultural non-recognition and stigma themes described under Domain 1, as well as distrust of healthcare providers, preferences for traditional or religious treatments, and the normalization of depression as an expected part of the immigrant experience. These barriers were self-reinforcing: when depression is not recognized as a health condition, there is little impetus to seek health services; when seeking services is stigmatized, non-recognition is reinforced.

#### 3.5.3. Intersectional Barriers

Several studies documented how intersecting identities—race, gender, immigration status, and nationality created compounded barriers to care. Olukotun and colleagues (S009) [41] provided the most vivid articulation of this phenomenon, with one participant stating, “I am Black, immigrant, I am undocumented. The Black community does not really address immigration. When you go to immigrant spaces, a lot of those organizations are mostly geared toward Latino immigrants, not really Black people… So it’s like, you’re a double minority on top of everything” (S009). This intersectional invisibility in which undocumented Black African immigrant women fell through the cracks of every institutional advocacy structure—represents a structural gap that directly contributed to isolation and impeded help-seeking.

#### 3.5.4. Community-Based Responses and Facilitators

Despite the formidable barriers described above, the literature also identified emergent community responses and potential facilitators for improving mental health support. Ludwig and Reed (S015) [47] documented a particularly compelling community-generated response: an African market where elderly Liberian women experienced relief from depressive symptoms through socialization, cultural practice, restored purpose, and the recovery of elder status lost through migration. This finding, along with Lipira and colleagues’ (S014) culturally adapted “African Market” multimedia intervention, a significant reduction in PHQ-8 scores with a medium effect size (d = 0.56, *p* = 0.01), suggests that culturally embedded community spaces may function as de facto mental health interventions.

Faith communities were identified as potential access points for mental health services in six studies (S003, S006, S008, S009, S010, S015), building on the centrality of religious coping documented above. The DiClemente-Bosco and colleagues (S006) study of Congolese refugees in Rhode Island demonstrated the Ubuntu philosophy of communal support, captured in the study’s title “I am because you are,” as a culturally resonant framework for mental health support. Education about depression was identified as a primary facilitator in multiple studies, most prominently by Ezeobele and colleagues (S008) [40], while Nakajima and colleagues (S016) [48] identified younger generation members as potential cultural bridges who could help translate between Western psychiatric concepts and traditional frameworks.

Notably, only one study in the entire review (S014) tested a formal intervention for depression. Lipira and colleagues developed a culturally adapted, peer-delivered multimedia intervention for East African immigrants with HIV, which produced a statistically significant reduction in PHQ-8 scores (*p* = 0.01, d = 0.56) after a single 90–120 min session. Although promising, this remains the sole intervention study identified in this review, leaving a substantial evidence gap between the comprehensive documentation of the problem and the development of targeted solutions.

### 3.6. Cross-Cutting Factors

#### 3.6.1. Gender

Gender emerged as a significant factor in 10 studies. The companion studies by Ezeobele and colleagues (S007, S008) provided the most direct gender comparison, finding that among Nigerian immigrants in Houston, cultural non-recognition of depression was consistent across genders. However, gender-specific mechanisms differed: men emphasized that acknowledging depression was incompatible with masculinity norms, while women described greater openness to discussing emotional distress in trusted settings. Paradoxically, Saasa and Miller (S019) found that being female was protective in their nationwide sample (β = −0.25, *p* < 0.05), with males reporting worse depression/anxiety symptoms—a finding that contradicts general population trends and aligns with the cultural masculinity barriers described qualitatively.

#### 3.6.2. Immigration Status and Documentation

Immigration status shaped depression experiences across the reviewed literature. The most dramatic evidence came from Olukotun and colleagues (S009) [41], the only study focused exclusively on undocumented immigrants, where undocumented status functioned not merely as a risk factor but as a root structural cause of depression through economic exploitation, social isolation, constant fear of deportation, and existential uncertainty. Kroll and colleagues (S011) [43] found that immigration status fears suppressed clinical disclosure among Somali refugees. Bentley et al. [34] (S002) identified a temporal dimension, finding that the relationship between depression and persistent multi-domain life distress became significant only after seven or more years of U.S. residence, suggesting a delayed-onset pattern that challenges the “healthy immigrant” narrative.

#### 3.6.3. Discrimination

Racial and anti-immigrant discrimination was identified as a predictor of depression in seven studies. Chirimwami and colleagues (S004) [36] found that participants with discrimination scores at or above the median were four to five times more likely to endorse depressive symptoms than those below the median. Saasa and Miller (S019) [51] found a significant bivariate correlation between discrimination and depression/anxiety (r = 0.559, *p* < 0.001), though this association was attenuated to non-significance (β = 0.14, *p* = 0.06) in the multivariate model—suggesting that discrimination’s effect may operate through mediating pathways including loneliness, substance use, and material deprivation. Importantly, loneliness significantly moderated the discrimination–mental health relationship (β = 0.43, *p* < 0.05), such that social connectedness buffered and loneliness amplified discrimination’s harmful effects.

Table 6 presents selected participant quotes and key findings organized by domain, illustrating the evidentiary basis for the themes synthesized above. Quotes are drawn from qualitative and mixed-methods studies. Quotation marks indicate direct participant or author language from the original studies.

Note added in revision in response to Reviewer 3’s request to “remove repetition of tables”: For the published version, Table 6 has been streamlined to retain only the most distinctive illustrative quotes per theme, removing entries whose content is already presented in detail in the narrative synthesis above (Section 3.2, Section 3.3, Section 3.4, Section 3.5 and Section 3.6). The retained quotes are those that (a) appear in direct participant voice rather than as paraphrased author statements, (b) introduce a finding not otherwise quoted in the body text, and (c) anchor a domain or cross-cutting theme. The full table is preserved here in the revised manuscript so reviewers can see the complete evidence base; per Reviewer 3’s guidance, the authors will work with editorial staff on the final trimmed version of Table 6 at the production stage.

### 3.7. Evidence Gaps and Directions for Future Research

The synthesis of 19 studies reveals a literature base that is growing but marked by significant gaps. Five priority areas for future research are identified below.

Measurement validation. No study in this review employed a depression instrument validated for Black African immigrant populations. Given the documented patterns of somatization, cultural non-recognition, and the linguistic absence of depression concepts, standard Western instruments may systematically misclassify or undercount depression in this population. Developing and validating culturally appropriate depression measures, potentially incorporating somatic symptom items and using concept-concordant language, is an urgent research priority.

Intervention development. Only one study (S014) tested a depression intervention, and it was limited to HIV-positive East Africans. Despite 18 studies documenting the scope of the problem, they have generated no evidence regarding solutions. Given the centrality of religious coping and social support, faith-based interventions and community-embedded approaches represent the most promising yet entirely untested pathways.

Longitudinal designs. All 19 studies employed cross-sectional or retrospective designs. Bentley and colleagues’ (S002) [34] finding of a seven-year threshold and Olukotun and colleagues’ (S009) [41] documentation of symptom fluctuation with political climate both urgently require longitudinal validation. Prospective studies tracking depression trajectories across the settlement period would clarify the temporal dynamics of depression in this population.

Underrepresented populations. The literature is heavily concentrated on Somali (seven studies) and Nigerian (two studies) populations, with minimal representation from Central, Southern, and Francophone African communities. Only one study focused on undocumented immigrants, one on older adults, and two included substantial male-focused data. Second-generation immigrants were entirely absent from the reviewed literature, despite evidence of generational shifts in depression perceptions (S016).

Structural determinants research. The qualitative evidence strongly implicates structural factors—immigration policy, documentation status, discrimination, and material deprivation—as drivers of depression, yet no study has quantitatively measured the impact of specific policies on depression outcomes. Natural experiments created by immigration policy changes could provide causal evidence for the structural determinants of depression documented qualitatively across this review.

## 4. Discussion

This scoping review mapped 19 studies examining how Black Sub-Saharan African immigrant adults in the United States perceive, report, and respond to depression. The synthesis reveals a population in which depression is widely experienced yet systematically rendered invisible through interlocking cultural, linguistic, structural, and institutional mechanisms. The findings carry significant implications for clinical practice, public health policy, and future research.

### 4.1. Depression as an Invisible Condition: A Multilevel Framework

Perhaps the most consequential finding of this review is that the invisibility of depression in Black Sub-Saharan African immigrant communities is not attributable to any single factor but to a reinforcing system operating across multiple levels. At the conceptual level, depression is not recognized as a discrete health condition in many cultural frameworks (S003, S007, S008, S015, S016). At the linguistic level, multiple African languages lack equivalent terminology for depression, anxiety, or mental health (S004, S008, S016). At the social level, profound stigma with concrete social consequences—including intergenerational impacts on marriage prospects (S008)—enforces silence. At the individual level, depression symptoms are normalized as expected consequences of the immigrant experience (S005, S007, S009, S015). At the institutional level, healthcare systems lack the culturally concordant tools and providers to detect depression even when patients present for care (S004, S011, S018). Each level reinforces the others, creating what can be described as an *architecture of invisibility* that renders depression simultaneously pervasive and undetectable.

This multilevel framework extends beyond existing theoretical models. While Kleinman’s [52] explanatory model framework accounts for cultural variation in illness understanding, and Bhui and colleagues’ [53] work on cultural idioms of distress addresses linguistic variation, neither fully captures the systemic nature of the invisibility documented here. The present findings suggest the need for an integrated framework—one that accounts for how conceptual non-recognition, linguistic absence, social sanction, individual normalization, and institutional inadequacy operate as a mutually reinforcing system.

### 4.2. Somatization as Cultural Communication, Not Pathology

The convergence of qualitative and quantitative evidence on somatic symptom expression represents one of the strongest findings of this review. The quantitative data from Nkimbeng and colleagues (S018) [50], showing sleep disturbance and fatigue endorsed at approximately twice the rate of depressed mood, were corroborated by rich qualitative accounts of depression embodied as hypertension (S015), headaches (S007, S008, S013), and generalized body pain (S001, S003). This triangulation across study designs strengthens the conclusion that somatization is not an artifact of measurement but a genuine cultural pattern of distress expression.

This finding is consistent with a substantial cross-cultural psychiatry literature documenting somatic emphasis in depression across non-Western populations [54,55,56,57]. However, the present review extends this literature in two important ways. First, it documents somatization specifically among Sub-Saharan African immigrants in the United States, a population for which somatic emphasis had been theorized but insufficiently documented. Second, it suggests that somatization in this population may be driven not merely by cultural expression norms but by the absence of conceptual frameworks for psychological distress—a qualitatively different mechanism than that documented in East Asian populations, where psychological distress is recognized but expression norms favor somatic communication [56].

The clinical implications are significant. Standard depression screening instruments such as the PHQ-8 and PHQ-9 weight psychological symptoms (depressed mood, anhedonia) as heavily as somatic symptoms, potentially under-detecting depression in populations where somatic symptoms predominate. The finding that “feeling down, depressed, or hopeless” was endorsed by only 9.5% of older African immigrants (S018) while sleep disturbance was endorsed by 20.3% suggests that screening protocols emphasizing psychological symptom items may systematically miss depression in this population.

### 4.3. The “Naming Gap” and Its Implications for Measurement

The convergent finding across 7 studies that multiple African languages lack direct equivalents for depression, anxiety, or mental health carries profound implications that extend beyond linguistics. When a condition cannot be named within a community’s conceptual framework, it cannot be recognized, communicated about, or sought treatment for through conventional channels. This “naming gap” operates as a fundamental barrier at every stage of the pathway from symptom experience to care access.

This finding resonates with Whorf’s [58] linguistic relativity hypothesis and more recent work by Wierzbicka [59,60] on the cultural specificity of emotion concepts. It also aligns with Kohrt and colleagues’ [61] documentation of translation challenges in global mental health measurement. However, the present review suggests the problem may be more fundamental than translation: if there is no source concept to translate from, translation-based approaches to cross-cultural measurement are inherently insufficient. What is needed instead are concept-concordant approaches that begin with local idioms and experiences rather than attempting to map Western psychiatric categories onto non-Western conceptual frameworks.

The practical strategy identified by Corley and Sabri (S005)—to use symptom-based rather than diagnostic language—and the deliberate reframing by Nevin and colleagues (S017) [49] to use “stress” and “mood” rather than “depression” during interviews both point toward workable clinical strategies. These approaches prioritize functional communication over diagnostic precision and may be more effective for initial screening and engagement than instrument-based approaches.

### 4.4. The Double-Edged Sword of Religious Coping

Religion emerged as the dominant response to depression across the reviewed literature, documented in 9 studies spanning both Christian and Muslim communities. However, the synthesis reveals a critically important duality. On one hand, religion provided hope, meaning, community, and a sense of purpose that directly countered the existential despair described by participants. On the other hand, religious frameworks could attribute depression to weak faith or spiritual failure (S016), religious coping was quantitatively associated with higher depression scores (S012), and reliance on religious healing could function as a mechanism of avoidance that delayed professional help-seeking (S003).

This duality echoes Pargament’s [62] distinction between positive religious coping (finding comfort, meaning, and support through faith) and negative religious coping (spiritual struggle, divine punishment attributions), a pattern subsequently documented across both Christian and Muslim communities [63], consistent with the cross-denominational pattern observed in this review. The present findings suggest that both forms may coexist within the same communities and even within the same individuals, complicating any simple recommendation to either leverage or bypass religious frameworks in intervention design. Rather, the evidence points toward collaboration with faith communities that preserves the protective elements of religious coping, including community belonging, meaning-making, and hope, while explicitly addressing the harmful potential of spiritual blame and treatment avoidance.

### 4.5. Structural Determinants and the Limits of Cultural Explanation

While much of the literature emphasizes cultural factors in depression perception and response, the present synthesis highlights the critical role of structural determinants that are irreducible to culture. Undocumented status (S009), discrimination (S004, S019), material deprivation (S019), environmental safety concerns (S019), and the sociopolitical climate (S005, S009) were all identified as significant predictors or drivers of depression. Olukotun and colleagues’ (S009) finding that depressive symptoms fluctuated with changes in political administration provides particularly compelling evidence that depression in this population is partly a product of the policy environment.

This finding cautions against explanatory frameworks that locate the “problem” of depression primarily within immigrant communities’ cultural beliefs or health literacy. While cultural factors shape how depression is perceived and expressed, the structural conditions that produce depression—economic exploitation, legal precarity, discrimination, social exclusion—are features of the receiving society, not the immigrant community. This distinction has important implications for intervention design: culturally adapted screening and treatment are necessary but insufficient without concurrent attention to the structural conditions that generate and sustain depression.

### 4.6. Implications for Clinical Practice

The findings of this review carry several actionable implications for healthcare providers serving Black Sub-Saharan African immigrant communities.

Screening approaches. Given the documented somatic emphasis and naming gap, providers should consider supplementing standard depression instruments with open-ended inquiry about somatic complaints, sleep disturbance, and fatigue. The use of symptom-based rather than diagnostic language (e.g., asking about specific experiences rather than “depression” or “mental health”) may improve detection. Proactive screening during primary care encounters is essential, as voluntary disclosure is extremely rare.

Provider education. Healthcare providers need foundational knowledge about the cultural frameworks documented in this review, including the sanity-insanity binary, spiritual explanatory models, and the intergenerational social consequences of mental health disclosure. This knowledge should inform clinical encounters without reducing individual patients to cultural stereotypes.

Structural competency. Beyond cultural competence, providers need structural competency [64]—an understanding of how immigration status, discrimination, and policy environments shape patients’ mental health and their ability to access care. For undocumented patients, the fear of deportation may be a more immediate barrier to disclosure than cultural beliefs about depression.

Faith community partnerships. Given the centrality of religion and the near-absence of formal mental health utilization, partnerships between healthcare systems and faith communities represent the most promising access pathway. Churches and mosques are among the few institutions that immigrant communities engage with voluntarily and trustingly. These partnerships must be designed to complement rather than compete with religious coping.

### 4.7. Implications for Policy

The evidence that immigration status functions as a root cause of depression (S009), that depression severity fluctuates with policy climate (S005, S009), and that undocumented immigrants are effectively locked out of mental health services points to policy interventions as potentially the most impactful lever for addressing depression in this population. Policies that reduce immigration-related precarity, extend healthcare access to undocumented individuals, protect against workplace exploitation, and counter xenophobic rhetoric may have direct mental health benefits. Additionally, investment in community-based mental health services that are accessible regardless of documentation status, linguistically concordant, and embedded within trusted community institutions is indicated.

### 4.8. Limitations

This scoping review has several limitations that should be considered when interpreting the findings.

Review-level limitations. First, consistent with scoping review methodology [27,30], no formal quality appraisal or risk of bias assessment was conducted for the included studies. While this approach is appropriate for mapping the breadth of evidence, it means that methodologically weak studies contributed equally to the synthesis alongside more rigorous designs. Second, the search was limited to English-language publications, potentially excluding relevant studies published in French or other languages that may have captured Francophone African immigrant experiences. Third, the search was restricted to studies conducted in the United States; findings may not generalize to Black Sub-Saharan African immigrants in other Western countries with different healthcare systems and immigration policies.

Literature-level limitations. The included literature was marked by significant methodological limitations that constrain the strength of conclusions. All 19 studies employed cross-sectional or retrospective designs, precluding causal inference. No study used a depression instrument validated for Black African immigrant populations, meaning prevalence estimates across the review are of uncertain accuracy. The literature is geographically concentrated, with 6 of 19 studies from the Seattle-Portland corridor, and population representation is uneven, with Somali immigrants appearing in 7 studies while Central and Southern African communities are minimally represented. Sample overlap between studies (S001/S002; S014/S017) means the effective number of unique participants is lower than the aggregate count suggests. Several studies omitted country-of-origin data for participant confidentiality, limiting the ability to disaggregate findings by nationality or cultural group.

Synthesis-level limitations. The heterogeneity of study designs, populations, depression measures, and outcome definitions precluded meta-analysis and limited the precision of cross-study comparisons. The thematic synthesis involved interpretive judgments in coding qualitative findings across diverse cultural contexts, and alternative interpretations of some themes are possible. Relevance ratings for included studies were determined through team review and consensus, with all reviewers contributing to final designations. While some subjectivity in rating assignment is inherent to scoping review methodology, the team-based approach reduced the risk of individual bias. Finally, the narrative synthesis necessarily simplified complex, culturally embedded phenomena into thematic categories that may not fully capture the nuance of individual studies.

Scope limitations. As a scoping review, this study aimed to map the breadth rather than synthesize the depth of evidence. Although grey literature sources were searched, including ProQuest Dissertations and Theses Global and Google Scholar, no additional studies were identified through these supplementary searches beyond those captured in the primary database search. It is possible that relevant community-based research that has not reached peer-reviewed publication remains unrepresented in this review, representing a potential limitation of the available evidence base rather than of the search strategy itself. The focus on depression as a specific condition may have excluded relevant studies examining broader constructs of psychological distress, well-being, or cultural syndromes that overlap with but do not directly address depression.

## 5. Conclusions

This scoping review of 19 studies reveals that depression among Black Sub-Saharan African immigrant adults in the United States is characterized by a paradox: it is both pervasive and invisible. Depression is widely experienced across diverse African immigrant communities, yet it is culturally unrecognized as a discrete health condition, linguistically unnamed in multiple African languages, expressed predominantly through somatic rather than psychological symptoms, socially sanctioned through profound stigma, individually normalized as an expected cost of immigration, and institutionally undetected by healthcare systems lacking culturally concordant approaches. The central conclusion of this review is therefore that the invisibility of depression in this population is not the product of any single cultural or individual factor but the cumulative effect of a mutually reinforcing architecture operating across conceptual, linguistic, somatic, social, and institutional levels.

Two further conclusions follow. First, structural determinants, including immigration status, discrimination, material deprivation, and the sociopolitical climate, are independently associated with depression in this population and are irreducible to cultural explanation. Second, the near-complete absence of intervention evidence, with only one such study among the 19 reviewed, constitutes the most urgent priority for future research. The reviewed literature documents the scope of this challenge with considerable richness across qualitative, quantitative, and mixed-methods designs. What it has not yet generated is an evidence base for solutions. With only one intervention study among the 19 included, and none outside of HIV-positive populations, the field faces an urgent need to translate its descriptive knowledge into culturally adapted, community-embedded, and structurally informed interventions. The most promising pathways identified by this review, including faith community partnerships, community-generated social spaces, symptom-based rather than diagnosis-based communication, and peer-delivered culturally adapted programs, await rigorous evaluation. Until such evidence is generated, a growing and underserved population will continue to experience depression in the shadows.

## Figures and Tables

**Table 1:** Relevance Rating Categories and Defining Criteria.

Rating	Defining Criteria
Very High	Study focused exclusively on Black Sub-Saharan African immigrants; addressed all three review domains substantively; provided rich, in-depth, theoretically generative findings central to the review question.
High	Study focused on Black Sub-Saharan African immigrants; addressed two or three review domains with substantive findings, or addressed one domain with exceptional depth.
Moderate-High	Study focused on Black Sub-Saharan African immigrants; addressed one or two domains with moderate depth, or addressed multiple domains with varying depth.
Moderate	Study provided findings relevant to the review but with one or more of the following limitations: narrower domain coverage, the African immigrant sample was part of a broader population, or findings were less developed.

Note. The three review domains are perceptions of depression, reporting and communication of depression, and responses to depression. The three assessment criteria (population focus, domain coverage, depth and richness of findings) were considered jointly rather than scored independently.

**Table 2:** Summary of Study Designs and Sample Characteristics.

ID	Authors, Year	Design	N	Population	Location	Measure	Relevance
S001	Bentley et al., 2011 [33]	Cross-sectional	74	Somali refugees	Seattle, WA	HSCL-25	Moderate
S002	Bentley et al., 2019 [34]	Cross-sectional	52	East African (Somali and Ethiopian) refugees	Seattle, WA	HSCL-25	Mod-High
S003	Boynton et al., 2010 [35]	Case study	1	Somali refugee	Seattle, WA	Clinical dx	High
S004	Chirimwami et al., 2025 [36]	Cross-sectional CBPR	385	Multi-SSA	Portland, OR	PHQ-8	High
S005	Corley & Sabri, 2021 [37]	Qualitative	39	Multi-SSA women (IPV)	DC/MD/VA	None	High
S006	DiClemente-Bosco et al., 2024 [38]	Qualitative CBPR	15	Congolese refugees	Providence, RI	None	Mod-High
S007	Ezeobele et al., 2019 [39]	Qualitative	18	Nigerian men	Houston, TX	None	High
S008	Ezeobele et al., 2010 [40]	Qualitative	19	Nigerian women	Houston, TX	None	Very High
S009	Olukotun et al., 2019 [41]	Qualitative	24	Undocumented SSA women	Midwest (88%)	None	High
S010	Kassa et al., 2025 [42]	Qualitative	45	Ethiopian	Ohio	None	Moderate
S011	Kroll et al., 2011 [43]	Chart review	600	Somali refugees	Minnesota	DSM-IV	Mod-High
S012	Leaman & Gee, 2012 [44]	Cross-sectional	131	Cameroon/Ethiopia	Mid-Atlantic	HSCL-25	Mod-High
S013	Lever et al., 2019 [45]	Mixed methods	13	W. African FGM/C	NYC	HSCL-25	Moderate
S014	Lipira et al., 2019 [46]	Mixed methods	25	E. African HIV+	Seattle, WA	PHQ-8	Mod-High
S015	Ludwig & Reed, 2016 [47]	Ethnographic	68	Liberian	Staten Island, NY	None	High
S016	Nakajima et al., 2023 [48]	Focus groups	47	Somali	Minnesota	None	Mod-High
S017	Nevin et al., 2018 [49]	Qualitative	20	E. African HIV+	Seattle, WA	None	Mod-High
S018	Nkimbeng et al., 2023 [50]	Cross-sectional	148	Older (50+) Africans	Baltimore-DC	PHQ-8	High
S019	Saasa & Miller, 2021 [51]	Cross-sectional	180	Multi-SSA	Nationwide	PHQ-4	Mod-High

Note. SSA = Sub-Saharan African; CBPR = Community-Based Participatory Research; IPV = intimate partner violence; FGM/C = female genital mutilation/cutting; HSCL-25 = Hopkins Symptom Checklist-25; PHQ = Patient Health Questionnaire; dx = diagnosis. Mod-High = Moderate-High relevance.

**Table 3:** Thematic Distribution Across Included Studies (N = 19); Primary findings (●) indicate the theme was a central or substantial finding of the study. Secondary findings (○) indicate the theme was mentioned, referenced, or addressed indirectly.

Theme	n	Studies with Primary Findings (●)	Studies with Secondary Findings (○)
**Domain 1: Perceptions of Depression**			
P1: Cultural non-recognition of depression	12	S003, S004, S005, S007, S008, S010, S015, S016	S001, S006, S009, S014
P2: Sanity–insanity binary (no spectrum)	2	S003, S016	-
P3: Spiritual/supernatural explanatory models	5	S003, S007, S008, S010, S016	-
P4: Depression as situational/contextual	7	S005, S009, S015	S003, S004, S006, S007, S008, S017
P5: Normalization of depression	7	S007, S008, S009, S015, S016	S005, S017
P6: Stigma and social consequences	14	S003, S005, S006, S007, S008, S010, S011, S014, S015, S016, S017	S004, S009, S018
P7: “Naming gap” (linguistic/conceptual absence)	7	S004, S005, S014, S016	S007, S008, S015
P8: Somatization as cultural framework	9	S001, S003, S007, S008, S013, S015, S018	S002, S016
**Domain 2: Reporting and Communication of Depression**			
R1: Somatic symptom emphasis in expression	9	S001, S003, S007, S008, S013, S015, S018	S002, S011
R2: Psychological/emotional language used	7	S005, S009, S017	S006, S008, S010, S014
R3: Non-disclosure/secrecy norms	8	S005, S009, S011, S014, S017	S001, S006, S007, S008
R4: Systematic underreporting concerns	11	S004, S005, S009, S011, S018, S019	S001, S002, S007, S008, S012
R5: Sociopolitical temporality of symptoms	2	S009	S005
R6: Quantitative prevalence data available	9	S001, S002, S004, S011, S012, S013, S014, S018, S019	-
**Domain 3: Responses to Depression**			
C1: Religious/spiritual coping	9	S003, S007, S008, S009, S010, S012, S016	S005, S006
C2: Social support networks	8	S005, S006, S009, S014, S015	S003, S007, S008, S010
C3: Traditional/indigenous treatments	4	S003, S007, S008, S010	-
C4: Low formal mental health utilization	9	S003, S008, S009, S014, S017	S005, S006, S007, S011
C5: Substance use as coping	2	S011, S019	-
C6: Structural barriers (insurance, cost, access)	11	S005, S006, S009, S010, S014	S001, S003, S004, S011, S017, S019
C7: Cultural barriers (stigma, beliefs, distrust)	15	S003, S004, S005, S006, S007, S008, S009, S010, S011, S015, S016, S017	S012, S013, S018
C8: Intersectional/immigration-specific barriers	7	S005, S009, S011, S014, S017	S006, S013
C9: Community-based responses	5	S006, S014, S015	S003, S004, S009
C10: Facilitators identified	10	S003, S006, S008, S009, S010, S014, S015	S004, S005, S016
**Cross-Cutting Factors**			
X1: Gender-specific findings	10	S005, S006, S007, S008, S009, S013, S019	S001, S003, S004
X2: Age/generational differences	4	S015, S016, S018	S011
X3: Immigration status as determinant	8	S006, S009, S011, S013, S014, S017	S001, S003, S004
X4: Discrimination/racism	7	S002, S004, S005, S019	S001, S006, S009, S012
X5: Acculturation/length of stay effects	5	S002, S015, S018	S004, S009
X6: Trauma/pre-migration experiences	10	S001, S002, S003, S005, S006, S011, S012, S013, S014, S017	-

Note. n = number of studies in which the theme was identified (primary or secondary). Themes are organized by domain. Study IDs correspond to Table 2. A theme was classified as “primary” if it was a central finding or substantial focus of the study, and “secondary” if it was mentioned, discussed indirectly, or supported by the study’s broader findings.

**Table 4:** Individual PHQ-8 Item Endorsement Rates Among Older African Immigrants (S018, N = 148).

PHQ-8 Item	% Endorsed	Symptom Type
Trouble falling/staying asleep or sleeping too much	20.3%	Somatic
Feeling tired or having little energy	18.9%	Somatic
Little interest or pleasure in doing things	15.5%	Psychological
Trouble concentrating	10.1%	Cognitive
Feeling down, depressed, or hopeless	9.5%	Psychological
Feeling bad about yourself/failure	7.4%	Psychological
Poor appetite or overeating	6.8%	Somatic
Moving/speaking slowly or fidgety/restless	2.0%	Psychomotor

Note. Percentage endorsing “more than half of the days” or “nearly every day.” Adapted from [50].

**Table 5:** Depression Prevalence Across Studies Using Standardized Measures.

ID	Authors	N	Measure	Cutoff	Prevalence	Mean (SD)	Key Context
S018	Nkimbeng, 2023 [50]	148	PHQ-8	≥10	8.1%	3.2 (3.9)	Older adults 50+; all with mobility limitations
S019	Saasa, 2021 [51]	180	PHQ-4	≥6	22% mod-sev	2.94 (3.4)	Combines depression + anxiety; nationwide
S004	Chirimwami, 2025 [36]	385	PHQ-8	≥10	7.9% moderate-severe	24.5% mild	CBPR; multi-country SSA; Portland, OR; symptoms elevated relative to 2022 US and US NHB norms
S014	Lipira, 2019 [46]	25	PHQ-8	≥5	72% mild+	Elevated	HIV+ only; pre-intervention
S011	Kroll, 2011 [43]	600	DSM-IV	Clinical	8–79%	N/A	Varies by age/sex; 25% denial rate
S012	Leaman, 2012 [44]	131	HSCL-25	≥1.75	94.7%	1.89	Exceptionally high; may reflect measurement
S013	Lever, 2019 [45]	13	HSCL-25	≥1.75	100%	N/R	All FGM/C survivors + asylum seekers

Note. Studies ordered from lowest to highest prevalence. PHQ = Patient Health Questionnaire; HSCL-25 = Hopkins Symptom Checklist-25; mod-sev = moderate to severe; N/R = not reported. No instrument was validated for African immigrant populations. Studies S001 [33] and S002 [34] both used the HSCL-25 but reported mean depression subscale scores only (S001: M = 20.4, SD = 6.3; S002: M = 19.67, SD = 5.89) rather than prevalence estimates and are therefore not included in this table. Per-item means for both samples (1.36 and 1.31, respectively) fell below the HSCL-25 clinical cutoff of ≥1.75, consistent with the subclinical elevation pattern documented across this literature.

**Table 6:** Selected Participant Quotes and Key Findings Organized by Domain.

Study ID	Author, Year	Theme	Quote/Key Finding	Significance for Review
**Domain 1: Perceptions of Depression**				
S007	Ezeobele et al., 2019 [39]	Cultural non-recognition	“Depression does not exist in the Nigerian culture.” Men further stated: “We do not believe in it… we were not raised to be depressed.”	Overarching theme among Nigerian men—cultural non-recognition reframed as personal/cultural failing
S008	Ezeobele et al., 2010 [40]	Cultural non-recognition	“Depression is Not Acceptable” (overarching theme). Women described: “In our culture, we are trained to endure… not complain.”	Foundational cultural stance among Nigerian women—endurance norm precludes recognition
S015	Ludwig & Reed, 2016 [47]	Cultural non-recognition	“While most interviewees did not identify them as mental health problems” (author observation). Participants experienced depression symptoms but had no framework for categorizing them as a health condition.	Liberians experienced depression but did not label it as such—the recognition gap in action
S004	Chirimwami et al., 2025 [36]	Naming gap	“There are no words for mental health or anxiety in Swahili or Somali.”	Community Advisory Board statement—linguistic absence as structural barrier to recognition, screening, and help-seeking
S014	Lipira et al., 2019 [46]	Naming gap	Participants needed help “just to give a name to what people are feeling.”	The naming gap is not merely linguistic but experiential—participants lacked a conceptual framework for labeling distress
S005	Corley & Sabri, 2021 [37]	Naming gap	Participants advised: “Don’t say mental health”—use symptom-based language instead when communicating about emotional distress.	Practical communication strategy with clinical implications for screening and provider communication
S003	Boynton et al., 2010 [35]	Spiritual models	Faduma attributed her distress to *jinn* (spirit possession) and sought Quran reading at the mosque as first-line treatment before psychiatric referral.	Somali spiritual explanatory model—religious healing as culturally sanctioned first response
S008	Ezeobele et al., 2010 [40]	Spiritual models	Women described *ogbanje* (water spirit/dual personality) as an explanation for depression. Fifty percent endorsed traditional/fetish treatments including consultations with *juju* priests.	Igbo-specific spiritual model—coexists with awareness of biomedical treatment
S016	Nakajima et al., 2023 [48]	Binary model/spiritual blame	Somali elders maintained a sane/insane binary with no intermediate category. Depression could be attributed to *“weak faith”* or spirit possession.	Religion as double-edged: faith provides resilience but also assigns blame for depression
S008	Ezeobele et al., 2010 [40]	Stigma	The marriage investigation system—families check for mental illness across generations before approving marriages. One example traced back to 1947.	Stigma operates intergenerationally through formal social mechanisms, affecting entire family networks
S005	Corley & Sabri, 2021 [37]	Stigma	“If you went to see a therapist, there was something wrong with you.”	Help-seeking itself is stigmatized as a marker of personal failure
S009	Olukotun et al., 2019 [41]	Normalization	Women “coped with their sadness and viewed it as a normal part of their experiences.”	Depression absorbed as an expected cost of undocumented life—normalization eliminates impetus for help-seeking
**Domain 2: Reporting and Communication of Depression**				
S018	Nkimbeng et al., 2023 [50]	Somatic emphasis	PHQ-8 item endorsement: sleep disturbance (20.3%) and fatigue (18.9%) endorsed at approximately twice the rate of “feeling down, depressed, or hopeless” (9.5%).	Strongest quantitative evidence of somatic > psychological symptom expression in the review
S015	Ludwig & Reed, 2016 [47]	Somatic embodiment	“When you are here, you have high blood pressure.” One man’s ailments reportedly “disappear” when visiting Liberia.	Depression embodied as chronic disease—location-dependent symptom expression challenges individual pathology models
S009	Olukotun et al., 2019 [41]	Psychological language	“Why am I here? What’s the purpose? Why am I wasting my time on this earth?”	Existential despair approaching suicidal ideation—undocumented women did use direct depression language when trusted
S009	Olukotun et al., 2019 [41]	Non-disclosure	Only 1 of 24 women (4.2%) had discussed mental health symptoms with any healthcare professional, despite over half reporting recurrent depression.	Lowest disclosure rate in the review—secrecy about immigration status extended to emotional distress
S011	Kroll et al., 2011 [43]	Non-disclosure	Twenty-five percent of Somali patients denied depression despite having received a clinical diagnosis, compared to 0% of non-Somali comparison patients.	Quantitative evidence of active denial—cultural non-recognition manifests as clinical non-disclosure
S009	Olukotun et al., 2019 [41]	Sociopolitical temporality	“When Obama was the president, I felt this sense of normalcy. The fear I feel now was not as pronounced.”	Novel finding—depression severity fluctuated with political administration, implicating policy as a structural determinant
**Domain 3: Responses to Depression**				
S009	Olukotun et al., 2019 [41]	Religious coping	“I would say Jesus. I just had to pray. And even with prayer, it was still hard.”	Faith as primary coping yet acknowledged as insufficient alone—captures the limits of religious coping
S009	Olukotun et al., 2019 [41]	Religious coping—purpose	Church volunteering gave “the purpose I needed. Just something to remind me that I am important.”	Religion providing existential purpose during depression—directly counters the meaninglessness of undocumented life
S012	Leaman & Gee, 2012 [44]	Religious coping—dual role	Religious coping was significantly correlated with *higher* depression scores, suggesting it may function as both comfort and avoidance of professional care.	Quantitative evidence of religion’s double-edged nature—protective and potentially harmful simultaneously
S009	Olukotun et al., 2019 [41]	Social support	“The people I tell are other Black people who are also undocumented versus other immigrants.”	Trust calibrated by shared identity and vulnerability—disclosure decisions are fine-grained and strategic
S014	Lipira et al., 2019 [46]	Confidentiality	Participants would accept peer support facilitators “as long as you’re not from my country.”	Confidentiality imperative extends even within immigrant communities— co-national proximity increases disclosure risk
S015	Ludwig & Reed, 2016 [47]	Community response	The African market—elderly Liberian women selling at an outdoor market experienced relief from depression through socialization, cultural practice, restored purpose, and recovery of elder status.	Community-generated, culturally embedded response—social space as de facto mental health intervention
S008	Ezeobele et al., 2010 [40]	Traditional treatment	Fifty percent of Nigerian women endorsed traditional/fetish treatments including *juju* priests, ground animal parts, and ritual pendants.	Traditional treatment coexists with awareness of biomedical approaches—not either/or but parallel systems
**Cross-Cutting Factors**				
S009	Olukotun et al., 2019 [41]	Intersectional invisibility	“I am Black, immigrant, I am undocumented. The Black community does not really address immigration… immigrant spaces are mostly geared toward Latino immigrants… So it’s like, you’re a double minority on top of everything.”	No institutional home for undocumented Black African women—falls through every advocacy structure
S017	Nevin et al., 2018 [49]	Community silence	“Depression is not very talked about in the African community.”	Silence framed as a community-level norm, not individual choice—compounds dual stigma of HIV + mental health
S019	Saasa & Miller, 2021 [51]	Gender paradox	Being female was protective (β = −0.25, *p* < 0.05), with males reporting worse depression/anxiety symptoms—contrary to general population trends.	Cultural masculinity norms may suppress recognition and help-seeking differently—male depression understudied

Note. Quotes in quotation marks represent direct participant or author language from the original studies. Non-quoted entries summarize key findings in the reviewer’s words. Studies are organized by domain; some studies appear multiple times when contributing to different themes. Study IDs correspond to Table 2.

## Data Availability

No new data were created or analyzed in this study. The protocol was registered with the Open Science Framework upon completion of the review and prior to journal submission (registration DOI: https://osf.io/g2aun; registered 9 April 2026).

## Supplementary Materials

The following supporting information can be downloaded at: https://www.mdpi.com/article/10.3390/nursrep16060196/s1, Supplementary File S1: Full Search Strategies for All Databases.

## Public Involvement Statement

No public involvement in any aspect of this research.

## Guidelines and Standards Statement

Because this article is a Scoping Review and does not involve original research or human participants, formal reporting guidelines for empirical studies were not applicable.

## Use of Artificial Intelligence

An artificial intelligence tool (Claude—Opus 4.8, Anthropic) was used to assist with refining the search terms and translating the search strategy across the six databases to accommodate platform-specific syntax conventions (e.g., MeSH versus subject headings, field tags, and Boolean operator formatting). All search strategies were reviewed and finalized by the authors in consultation with a health sciences librarian, pilot-tested before full implementation, and verified for accuracy and completeness. The AI tool played no role in study selection, data extraction, data interpretation, or analytical judgment.
