# Supplementary material for: Perceptions, Reporting, and Responses to Depression Among Black Sub-Saharan African Immigrant Adults in the United States: A Scoping Review
Source: Nurs Rep. 2026 Jun 8;16(6):196. doi: 10.3390/nursrep16060196 (PMC13306222; doi:10.3390/nursrep16060196)
Supplement: Supplementary file 1 [file nursrep-16-00196-s001.zip › nursrep-4318650-supplementary.pdf]

Supplementary File 1

Full Search Strategies for All Databases

Perceptions, Reporting, and Responses to Depression Among Black Sub-Saharan African Immigrant Adults in the United States: A Scoping Review

Overview

This supplementary file provides the complete, reproducible search strategies used for each of the six electronic databases searched for this scoping review. Searches were conducted to identify empirical studies published between January 2000 and January 2026 examining perceptions, reporting, and responses to depression among Black Sub-Saharan African immigrant adults in the United States.

The search strategy combined four concept blocks using Boolean operators:

Concept 1: Sub-Saharan African immigrants, including specific country names and terms such as African immigrant, African refugee, and Black African.

Concept 2: Depression and mental health.

Concept 3: Perceptions, help-seeking, barriers, reporting, and responses.

Concept 4 (geographic limiter): United States.

Medical Subject Headings (MeSH) and database-specific controlled vocabulary were utilized where applicable. Free-text terms were searched in title and abstract fields. Truncation (denoted by an asterisk, *) was used to capture variant word endings (e.g., immigrant* retrieves immigrant, immigrants). Phrase searching was used with double quotation marks for multi-word terms. The search strategy was developed in consultation with a health sciences librarian and pilot-tested before full implementation.

Summary of Records Retrieved by Database

Database	Records Retrieved	Platform / Interface
CINAHL	752	EBSCOhost
Web of Science Core Collection	220	Clarivate Web of Science
PubMed	104	NCBI / NLM
Scopus	41	Elsevier Scopus
PsycINFO	35	EBSCOhost

Database	Records Retrieved	Platform / Interface
Sociological Abstracts	30	ProQuest
**Total**	**1,182**	

Note. Supplementary searches of ProQuest Dissertations and Theses Global and Google Scholar were also conducted to identify grey literature; these supplementary searches yielded no additional records beyond those identified through database searching.

1. PubMed

Platform: NCBI / U.S. National Library of Medicine

Date searched: January 2026

Records retrieved: 104

Field tags used: [MeSH] = Medical Subject Heading; [tiab] = title/abstract; [tw] = text word; [dp] = date of publication; [la] = language

Search strategy

#1 "Africa South of the Sahara"[MeSH] OR "Sub-Saharan African"[tiab] OR "Sub Saharan African"[tiab] OR "Black African*"[tiab] OR Nigerian*[tiab] OR Ethiopian*[tiab] OR Ghanaian*[tiab] OR Kenyan*[tiab] OR Somali*[tiab] OR Liberian*[tiab] OR Sudanese[tiab] OR Eritrean*[tiab] OR Ugandan*[tiab] OR Tanzanian*[tiab] OR Cameroonian*[tiab] OR Senegalese[tiab] OR Congolese[tiab] OR Zimbabwean*[tiab] OR "Sierra Leonean*"[tiab] OR Rwandan*[tiab] OR Burundian*[tiab] OR Malian*[tiab] OR "Ivorian*"[tiab]

#2 "Emigrants and Immigrants"[MeSH] OR "Refugees"[MeSH] OR "Transients and Migrants"[MeSH] OR immigrant*[tiab] OR refugee*[tiab] OR migrant*[tiab] OR "asylum seeker*"[tiab] OR "foreign-born"[tiab] OR "foreign born"[tiab] OR "African immigrant*"[tiab] OR "African refugee*"[tiab] OR "African migrant*"[tiab] OR "African-born"[tiab] OR "African born"[tiab]

#3 #1 AND #2

#4 "Depression"[MeSH] OR "Depressive Disorder"[MeSH] OR "Depressive Disorder, Major"[MeSH] OR "Mental Health"[MeSH] OR "Mental Disorders"[MeSH] OR "Affective Symptoms"[MeSH] OR depress*[tiab] OR "mental health"[tiab] OR "mental illness"[tiab] OR "mental disorder*"[tiab] OR "psychological distress"[tiab] OR "emotional distress"[tiab] OR "mood disorder*"[tiab]

#5 "Help-Seeking Behavior"[MeSH] OR "Patient Acceptance of Health Care"[MeSH] OR "Health Knowledge, Attitudes, Practice"[MeSH] OR "Social Stigma"[MeSH] OR "Adaptation, Psychological"[MeSH] OR "Culture"[MeSH] OR perception*[tiab] OR perceive*[tiab] OR belief*[tiab] OR attitude*[tiab] OR "help-seeking"[tiab] OR "help seeking"[tiab] OR "treatment-seeking"[tiab] OR "treatment seeking"[tiab] OR "service utilization"[tiab] OR "service use"[tiab] OR barrier*[tiab] OR facilitator*[tiab] OR stigma*[tiab] OR "explanatory model*"[tiab] OR "illness perception*"[tiab] OR

experience*[tiab] OR coping[tiab] OR response*[tiab] OR reporting[tiab] OR disclosure[tiab] OR somat*[tiab] OR "cultural belief*"[tiab]

#6 "United States"[MeSH] OR "United States"[tiab] OR USA[tiab] OR "U.S."[tiab] OR American*[tiab]

#7 #3 AND #4 AND #5 AND #6

#8 #7 AND ("2000/01/01"[dp] : "2026/01/31"[dp]) AND English[la]

2. PsycINFO

Platform: EBSCOhost

Date searched: January 2026

Records retrieved: 35

Field tags used: DE = exact subject heading; TI = title; AB = abstract; KW = author-supplied keywords

Search strategy

S1 DE "Africa" OR DE "Sub-Saharan Africa" OR TI ("Sub-Saharan African" OR "Sub Saharan African" OR "Black African*" OR Nigerian* OR Ethiopian* OR Ghanaian* OR Kenyan* OR Somali* OR Liberian* OR Sudanese OR Eritrean* OR Ugandan* OR Tanzanian* OR Cameroonian* OR Senegalese OR Congolese OR Zimbabwean* OR "Sierra Leonean*" OR Rwandan* OR Burundian* OR Malian*) OR AB ("Sub-Saharan African" OR "Sub Saharan African" OR "Black African*" OR Nigerian* OR Ethiopian* OR Ghanaian* OR Kenyan* OR Somali* OR Liberian* OR Sudanese OR Eritrean* OR Ugandan* OR Tanzanian* OR Cameroonian* OR Senegalese OR Congolese OR Zimbabwean* OR "Sierra Leonean*" OR Rwandan* OR Burundian* OR Malian*)

S2 DE "Immigration" OR DE "Immigrants" OR DE "Refugees" OR TI (immigrant* OR refugee* OR migrant* OR "asylum seeker*" OR "foreign-born" OR "foreign born" OR "African immigrant*" OR "African refugee*" OR "African migrant*" OR "African-born") OR AB (immigrant* OR refugee* OR migrant* OR "asylum seeker*" OR "foreign-born" OR "foreign born" OR "African immigrant*" OR "African refugee*" OR "African migrant*" OR "African-born")

S3 S1 AND S2

S4 DE "Major Depression" OR DE "Depression (Emotion)" OR DE "Mental Health" OR DE "Mental Disorders" OR DE "Affective Disorders" OR TI (depress* OR "mental health" OR "mental illness" OR "mental disorder*" OR "psychological distress" OR "emotional distress" OR "mood disorder*") OR AB (depress* OR "mental health" OR "mental illness" OR "mental disorder*" OR "psychological distress" OR "emotional distress" OR "mood disorder*")

S5 DE "Help Seeking Behavior" OR DE "Health Care Utilization" OR DE "Attitudes Toward Mental Health" OR DE "Stigma" OR DE "Coping Behavior" OR DE "Cultural

Sensitivity" OR TI (perception* OR perceive* OR belief* OR attitude* OR "help-seeking" OR "help seeking" OR "treatment-seeking" OR "service utilization" OR barrier* OR facilitator* OR stigma* OR "explanatory model*" OR "illness perception*" OR experience* OR coping OR response* OR reporting OR disclosure OR somat* OR "cultural belief*") OR AB (perception* OR perceive* OR belief* OR attitude* OR "help-seeking" OR "help seeking" OR "treatment-seeking" OR "service utilization" OR barrier* OR facilitator* OR stigma* OR "explanatory model*" OR "illness perception*" OR experience* OR coping OR response* OR reporting OR disclosure OR somat* OR "cultural belief*")

S6 DE "United States" OR TI ("United States" OR USA OR "U.S." OR American*) OR AB ("United States" OR USA OR "U.S." OR American*)

S7 S3 AND S4 AND S5 AND S6

S8 S7 limited to: Publication Date 2000-01-01 to 2026-01-31; Language English; Peer Reviewed

3. CINAHL

Platform: EBSCOhost

Date searched: January 2026

Records retrieved: 752

Field tags used: MH = exact CINAHL subject heading; TI = title; AB = abstract

Search strategy

S1 (MH "Africa, Central") OR (MH "Africa, Eastern") OR (MH "Africa, Western") OR (MH "Africa, Southern") OR TI ("Sub-Saharan African" OR "Sub Saharan African" OR "Black African*" OR Nigerian* OR Ethiopian* OR Ghanaian* OR Kenyan* OR Somali* OR Liberian* OR Sudanese OR Eritrean* OR Ugandan* OR Tanzanian* OR Cameroonian* OR Senegalese OR Congolese OR Zimbabwean* OR "Sierra Leonean*" OR Rwandan* OR Burundian* OR Malian*) OR AB ("Sub-Saharan African" OR "Sub Saharan African" OR "Black African*" OR Nigerian* OR Ethiopian* OR Ghanaian* OR Kenyan* OR Somali* OR Liberian* OR Sudanese OR Eritrean* OR Ugandan* OR Tanzanian* OR Cameroonian* OR Senegalese OR Congolese OR Zimbabwean* OR "Sierra Leonean*" OR Rwandan* OR Burundian* OR Malian*)

S2 (MH "Immigrants") OR (MH "Refugees") OR (MH "Transients and Migrants") OR TI (immigrant* OR refugee* OR migrant* OR "asylum seeker*" OR "foreign-born" OR "foreign born" OR "African immigrant*" OR "African refugee*" OR "African migrant*") OR AB (immigrant* OR refugee* OR migrant* OR "asylum seeker*" OR "foreign-born" OR "foreign born" OR "African immigrant*" OR "African refugee*" OR "African migrant*")

S3 S1 AND S2

S4 (MH "Depression") OR (MH "Depression, Postpartum") OR (MH "Mental Health") OR (MH "Mental Disorders") OR (MH "Affective Disorders") OR TI (depress* OR "mental health" OR "mental illness" OR "mental disorder*" OR "psychological distress" OR "emotional distress" OR "mood disorder*") OR AB (depress* OR "mental health" OR "mental illness" OR "mental disorder*" OR "psychological distress" OR "emotional distress" OR "mood disorder*")

S5 (MH "Help Seeking Behavior") OR (MH "Health Services Accessibility") OR (MH "Attitude to Mental Illness") OR (MH "Stigma") OR (MH "Adaptation, Psychological") OR (MH "Cultural Competence") OR TI (perception* OR perceive* OR belief* OR attitude* OR

"help-seeking" OR "help seeking" OR "treatment-seeking" OR "service utilization" OR barrier* OR facilitator* OR stigma* OR "explanatory model*" OR "illness perception*" OR experience* OR coping OR response* OR reporting OR disclosure OR somat* OR "cultural belief*") OR AB (perception* OR perceive* OR belief* OR attitude* OR "help-seeking" OR "help seeking" OR "treatment-seeking" OR "service utilization" OR barrier* OR facilitator* OR stigma* OR "explanatory model*" OR "illness perception*" OR experience* OR coping OR response* OR reporting OR disclosure OR somat* OR "cultural belief*")

S6 (MH "United States") OR TI ("United States" OR USA OR "U.S." OR American*) OR AB ("United States" OR USA OR "U.S." OR American*)

S7 S3 AND S4 AND S5 AND S6

S8 S7 limited to: Publication Date 2000-2026; Language English; Peer Reviewed

4. Web of Science Core Collection

Platform: Clarivate Web of Science

Date searched: January 2026

Records retrieved: 220

Field tags used: TS = topic (searches title, abstract, author keywords, and Keywords Plus); PY = publication year; LA = language

Search strategy

#1 TS=("Sub-Saharan African" OR "Sub Saharan African" OR "Black African*" OR Nigerian* OR Ethiopian* OR Ghanaian* OR Kenyan* OR Somali* OR Liberian* OR Sudanese OR Eritrean* OR Ugandan* OR Tanzanian* OR Cameroonian* OR Senegalese OR Congolese OR Zimbabwean* OR "Sierra Leonean*" OR Rwandan* OR Burundian* OR Malian*)

#2 TS=(immigrant* OR refugee* OR migrant* OR "asylum seeker*" OR "foreign-born" OR "foreign born" OR "African immigrant*" OR "African refugee*" OR "African migrant*" OR "African-born")

#3 #1 AND #2

#4 TS=(depress* OR "mental health" OR "mental illness" OR "mental disorder*" OR "psychological distress" OR "emotional distress" OR "mood disorder*" OR "major depressive disorder")

#5 TS=(perception* OR perceive* OR belief* OR attitude* OR "help-seeking" OR "help seeking" OR "treatment-seeking" OR "treatment seeking" OR "service utilization" OR "service use" OR barrier* OR facilitator* OR stigma* OR "explanatory model*" OR "illness perception*" OR experience* OR coping OR response* OR reporting OR disclosure OR somat* OR "cultural belief*")

#6 TS=("United States" OR USA OR "U.S." OR American*)

#7 #3 AND #4 AND #5 AND #6

#8 #7 AND PY=(2000-2026) AND LA=(English)

5. Scopus

Platform: Elsevier Scopus

Date searched: January 2026

Records retrieved: 41

Field tags used: TITLE-ABS-KEY = title, abstract, and keywords combined; PUBYEAR = publication year; LANGUAGE = language of publication

Search strategy

#1 TITLE-ABS-KEY("Sub-Saharan African" OR "Sub Saharan African" OR "Black African*" OR Nigerian* OR Ethiopian* OR Ghanaian* OR Kenyan* OR Somali* OR Liberian* OR Sudanese OR Eritrean* OR Ugandan* OR Tanzanian* OR Cameroonian* OR Senegalese OR Congolese OR Zimbabwean* OR "Sierra Leonean*" OR Rwandan* OR Burundian* OR Malian*)

#2 TITLE-ABS-KEY(immigrant* OR refugee* OR migrant* OR "asylum seeker*" OR "foreign-born" OR "foreign born" OR "African immigrant*" OR "African refugee*" OR "African migrant*" OR "African-born")

#3 #1 AND #2

#4 TITLE-ABS-KEY(depress* OR "mental health" OR "mental illness" OR "mental disorder*" OR "psychological distress" OR "emotional distress" OR "mood disorder*")

#5 TITLE-ABS-KEY(perception* OR perceive* OR belief* OR attitude* OR "help-seeking" OR "help seeking" OR "treatment-seeking" OR "service utilization" OR barrier* OR facilitator* OR stigma* OR "explanatory model*" OR "illness perception*" OR experience* OR coping OR response* OR reporting OR disclosure OR somat* OR "cultural belief*")

#6 TITLE-ABS-KEY("United States" OR USA OR "U.S." OR American*)

#7 #3 AND #4 AND #5 AND #6

#8 #7 AND PUBYEAR > 1999 AND PUBYEAR < 2027 AND LANGUAGE(english)

6. Sociological Abstracts

Platform: ProQuest

Date searched: January 2026

Records retrieved: 30

Field tags used: SU = subject; TI = title; AB = abstract; YR = publication year

Search strategy

S1 SU("Sub-Saharan Africa" OR "Africans") OR TI("Sub-Saharan African" OR "Sub Saharan African" OR "Black African*" OR Nigerian* OR Ethiopian* OR Ghanaian* OR Kenyan* OR Somali* OR Liberian* OR Sudanese OR Eritrean* OR Ugandan* OR Tanzanian* OR Cameroonian* OR Senegalese OR Congolese OR Zimbabwean* OR "Sierra Leonean*" OR Rwandan* OR Burundian* OR Malian*) OR AB("Sub-Saharan African" OR "Sub Saharan African" OR "Black African*" OR Nigerian* OR Ethiopian* OR Ghanaian* OR Kenyan* OR Somali* OR Liberian* OR Sudanese OR Eritrean* OR Ugandan* OR Tanzanian* OR Cameroonian* OR Senegalese OR Congolese OR Zimbabwean* OR "Sierra Leonean*" OR Rwandan* OR Burundian* OR Malian*)

S2 SU("Immigrants" OR "Refugees" OR "Migration") OR TI(immigrant* OR refugee* OR migrant* OR "asylum seeker*" OR "foreign-born" OR "African immigrant*" OR "African refugee*") OR AB(immigrant* OR refugee* OR migrant* OR "asylum seeker*" OR "foreign-born" OR "African immigrant*" OR "African refugee*")

S3 S1 AND S2

S4 SU("Depression (Psychology)" OR "Mental Health" OR "Mental Disorders") OR TI(depress* OR "mental health" OR "mental illness" OR "mental disorder*" OR "psychological distress" OR "emotional distress") OR AB(depress* OR "mental health" OR "mental illness" OR "mental disorder*" OR "psychological distress" OR "emotional distress")

S5 SU("Help Seeking Behavior" OR "Stigma" OR "Coping" OR "Cultural Beliefs") OR TI(perception* OR perceive* OR belief* OR attitude* OR "help-seeking" OR "help seeking" OR "service utilization" OR barrier* OR facilitator* OR stigma* OR "explanatory model*" OR experience* OR coping OR response* OR reporting OR disclosure OR somat*) OR AB(perception* OR perceive* OR belief* OR attitude* OR "help-seeking" OR "help

seeking" OR "service utilization" OR barrier* OR facilitator* OR stigma* OR "explanatory model*" OR experience* OR coping OR response* OR reporting OR disclosure OR somat*)

S6 SU("United States") OR TI("United States" OR USA OR American*) OR AB("United States" OR USA OR American*)

S7 S3 AND S4 AND S5 AND S6

S8 S7 limited to: YR(2000-2026); Language English; Peer Reviewed

Notes on Search Strategy Implementation

Translation across databases. Search strings were translated for each database to accommodate platform-specific syntax conventions while preserving the underlying conceptual structure. Where exact equivalents for a controlled vocabulary term were unavailable, the closest available subject heading was used in combination with free-text terms to maintain sensitivity.

Country and nationality terms. Country-of-origin terms were selected to reflect the major sending countries of Sub-Saharan African immigrants to the United States and the country-of-origin distribution observed in preliminary scoping searches. Terms for both nationality (e.g., Nigerian) and country (e.g., Nigeria) were tested, with nationality terms providing higher precision and retained in the final strategy.

Geographic limiter. The United States geographic limiter was applied as a separate concept block rather than as a database filter to ensure that studies indexed without a geographic descriptor but conducted in the United States were captured. Studies returned by the search but conducted outside the United States were excluded at the screening stage.

Grey literature. Supplementary searches of ProQuest Dissertations and Theses Global and Google Scholar were conducted using simplified versions of the strategies above. These supplementary searches yielded no additional unique records beyond those identified through the primary database searches.

Date limits. Searches were limited to publications dated January 1, 2000 through January 31, 2026, consistent with the review's eligibility criteria. The 2000 start date was selected to capture the period of substantial demographic growth in the U.S. Black Sub-Saharan African immigrant population.

Language. Searches were limited to English-language publications, consistent with the review's eligibility criteria.

Reproducibility. All search strategies presented in this supplementary file are reproducible as written. Researchers wishing to update or replicate this search are advised that subject heading vocabularies are revised periodically by database providers; users should verify current controlled vocabulary terms before re-executing the strategies.
